# Genome-wide association multi-locus and multi-variate linear mixed models reveal two linked loci with major effects on partial resistance of apricot to bacterial canker

**DOI:** 10.1186/s12870-019-1631-3

**Published:** 2019-01-21

**Authors:** Mariem Omrani, Morgane Roth, Guillaume Roch, Alain Blanc, Cindy E. Morris, Jean-Marc Audergon

**Affiliations:** 1 INRA, UR1052 Génétique et Amélioration des Fruits et Légumes, Centre de Recherche PACA, Montfavet, France; 2 INRA, UR407 Pathologie Végétale, Centre de Recherche PACA, Montfavet, France; 3 ENGREF, AgroParisTech, Paris, France; 4 CEP Innovation, Lyon, France; 5 Present Address: Agroscope, Research Division Plant Breeding, Wädenswil, Switzerland

**Keywords:** GWAS, *Prunus*, Partial resistance, Bacterial canker, *Pseudomonas syringae*, Candidate genes, Apricot, Multi-locus mixed model, Multi-variate mixed model, Linkage disequilibrium

## Abstract

**Background:**

Diseases caused by *Pseudomonas syringae* (*Ps*) are recognized as the most damaging factors in fruit trees with a significant economic and sanitary impact on crops. Among them, bacterial canker of apricot is exceedingly difficult to control due to a lack of efficient prophylactic measures. Several sources of partial resistance have been identified among genetic resources but the underlying genetic pattern has not been elucidated thus far. In this study, we phenotyped bacterial canker susceptibility in an apricot core-collection of 73 accessions over 4 years by measuring canker and superficial browning lengths issued from artificial inoculations in the orchard.

In order to investigate the genetic architecture of partial resistance, we performed a genome-wide association study using best linear unbiased predictors on genetic (*G*) and genetic x year (*G* × *Y*) interaction effects extracted from linear mixed models.

Using a set of 63,236 single-nucleotide polymorphism markers genotyped in the germplasm over the whole genome, multi-locus and multi-variate mixed models aimed at mapping the resistance while controlling for relatedness between individuals.

**Results:**

We detected 11 significant associations over 7 candidate loci linked to disease resistance under the two most severe years. Colocalizations between *G* and *G* × *Y* terms indicated a modulation on allelic effect depending on environmental conditions. Among the candidate loci, two loci on chromosomes 5 and 6 had a high impact on both canker length and superficial browning, explaining 41 and 26% of the total phenotypic variance, respectively. We found unexpected long-range linkage disequilibrium (LD) between these two markers revealing an inter-chromosomal LD block linking the two underlying genes. This result supports the hypothesis of a co-adaptation effect due to selection through population demography. Candidate genes annotations suggest a functional pathway involving abscisic acid, a hormone mainly known for mediating abiotic stress responses but also reported as a potential factor in plant-pathogen interactions.

**Conclusions:**

Our study contributed to the first detailed characterization of the genetic determinants of partial resistance to bacterial canker in a *Rosaceae* species. It provided tools for fruit tree breeding by identifying progenitors with favorable haplotypes and by providing major-effect markers for a marker-assisted selection strategy.

**Electronic supplementary material:**

The online version of this article (10.1186/s12870-019-1631-3) contains supplementary material, which is available to authorized users.

## Background

For decades, breeding programs have notably focused on the introgression of major resistant genes into crop cultivars. However multiple episodes of resistance breakdown have led research efforts to target polygenic (quantitative or partial) resistances [1]. Although breeding strategies relying on pyramiding quantitative trait loci (QTLs) on top of monogenic genes might be costly and time-consuming, the long-term efficiency and sustainability of QTLs have been assumed if they are deployed parsimoniously in both space and time [2, 3]. Resistance sustainability has a particular interest for perennial woody crops considering their long generation time and life-span in orchards, and overall the long-term commitment to their breeding schemes.

Within the *Prunus* genus, apricot (*Prunus armeniaca* L.) is one of the most popular and typical crops of the Mediterranean Basin which provides 49% of the total production to the world market [4]. Among the biotic stresses affecting apricot crop durability and in a broader way stone fruit species, bacterial canker, caused by ubiquitous bacteria in the species complex *Pseudomonas syringae* (*Ps*), is one of the most damaging. This disease could potentially lead to the death of trees in the orchard, especially young trees within their five first years after planting. Three main groups - *Ps* pv*. syringae, Ps viridiflava* [5] and *Ps* pv*. morsprunorum* [6], associated with phylogroups 2, 7 and 3, respectively [7], have been historically associated with the disease on apricot. The symptoms affect aerial organs, resulting in lesions and shot holes in leaves, bud and blossom dieback, and apical necrosis, potentially leading to more severe symptoms when bacteria get to the vessels and spread through the vascular system [8, 9]. When the infection is systemic, cankers corresponding to necrosis and flattening of external tissues linked to a dissymmetric growth of the cambium in the spring, are visible on branches and scaffold limbs [10]. Several abiotic factors relying on soil and climatic conditions can favor bacterial canker severity. Among them, cold winter temperatures and especially high frequency of frost-defrost episodes have been highlighted as major factors favoring bacterial canker [11, 12]. Integrated management practices such as the use of resistant and soil-adapted rootstock material and grafting at a tall height could lower bacterial canker incidence in orchards [13, 14]. These cultural recommendations are nevertheless, technically challenging for orchard management and provide only a partial protection in orchards.

Therefore, the development of partially resistant cultivars seems to be a promising measure in addition to preventive practices to assure orchard durability. In this context, research efforts have focused on screening apricot genetic resources both under natural infections or controlled inoculations. Several genetic backgrounds with partial resistance to bacterial canker have been identified: ‘Bakour’ [14], ‘Hâtif Colomer’, ‘Luizet’, ‘Palsteyn’ [15] and ‘Orangered’ [16]. More globally, similar research initiatives have been conducted to phenotypically characterize differential susceptibilities to bacterial canker in *Prunus* rootstocks [17], sweet cherry [17–19] and plum [20], but to date the question of the genetic determinants of partial resistance to bacterial canker from the natural diversity in *Rosaceae* fruit trees has never been investigated.

Recent progress of high-throughput-sequencing technologies fostering the discovery of thousands and even millions of single nucleotide polymorphisms (SNPs) over whole genomes has opened up new opportunities to address the underlying architecture of quantitative traits [21]. Genome-Wide Association Studies (GWAS) have been extensively and successfully used in plant breeding thanks to the advancement of these novel genomics-based approaches [22]. GWAS enable mapping of QTLs and genes affecting trait variation in a wide collection of mostly unrelated individuals usually sampled from wild relative populations, breeding cultivars and landraces. This is in stark contrast to traditional linkage mapping methods that require the establishment of segregating populations beforehand [23, 24]. Association studies benefit from the numerous recombination events, which have occurred through species demographic history, and rely on linkage disequilibrium (LD) caused in part by selection and population structure, to exploit natural allelic diversity and identify links between markers and causal loci underlying the trait of interest [25–27].

GWAS turns out to be a particularly suitable approach for plants and especially for perennial crops since costs associated with making and maintaining large progenies in the orchard can be spared. Therefore, the use of GWAS has gradually become more widespread for determining the genetic basis of variation in complex traits in *Rosaceae* fruit species [28–30]. More particularly, the apricot genome displays many advantages for GWAS applications due to: (i) its diploidy (2n = 16) and small size (294 Mb/n) [31], (ii) its high level of heterozygosity resulting from a general outcrossing mating system [32] and (iii) its high nucleotide diversity related both to the early seed propagation [33] and geographically broad distribution of the germplasm with a diversification from Central Asia [34, 35]. Moreover, the apricot genome has been characterized with a very fast LD decay within 100 base pairs (bp) in a large genetic diversity panel [30], allowing association mapping with a very precise resolution conditionally upon the use of a high density of markers [36].

In the present study, we investigate the underlying genetic architecture of partial resistance to bacterial canker in apricot using an association approach. The specific objectives of this research are to (i) identify the genetic and environmental components of bacterial canker susceptibility using a core-collection of 73 apricot accessions that has been phenotyped in the orchard over a 4-year campaign, (ii) develop an appropriate genome-wide association methodology taking into account the species genetic architecture and the stratification of our population set, and (iii) identify candidate genes and decipher the molecular basis of polygenic resistance to bacterial canker.

## Results

### Phenotypic variance decomposition and heritability of partial resistance to bacterial canker

Considering differences between all controls and inoculated shoots regardless of the genotype effect, a highly significant effect of bacterial inoculation was observed (Wilcoxon unilateral t.test *p* < 2.2E-16) for *lgc* whatever the year and for *bs* in 2013 and 2015 (lesser extent in 2014 *p* < 9.77E-12 and 2016 *p* < 9.06E-06). In the variance analysis, both branch height and shoot diameter had non-significant effects on the variation for the two phenotypes and were thus discarded from the multi-year and within-year models.

**Fig. 1:**
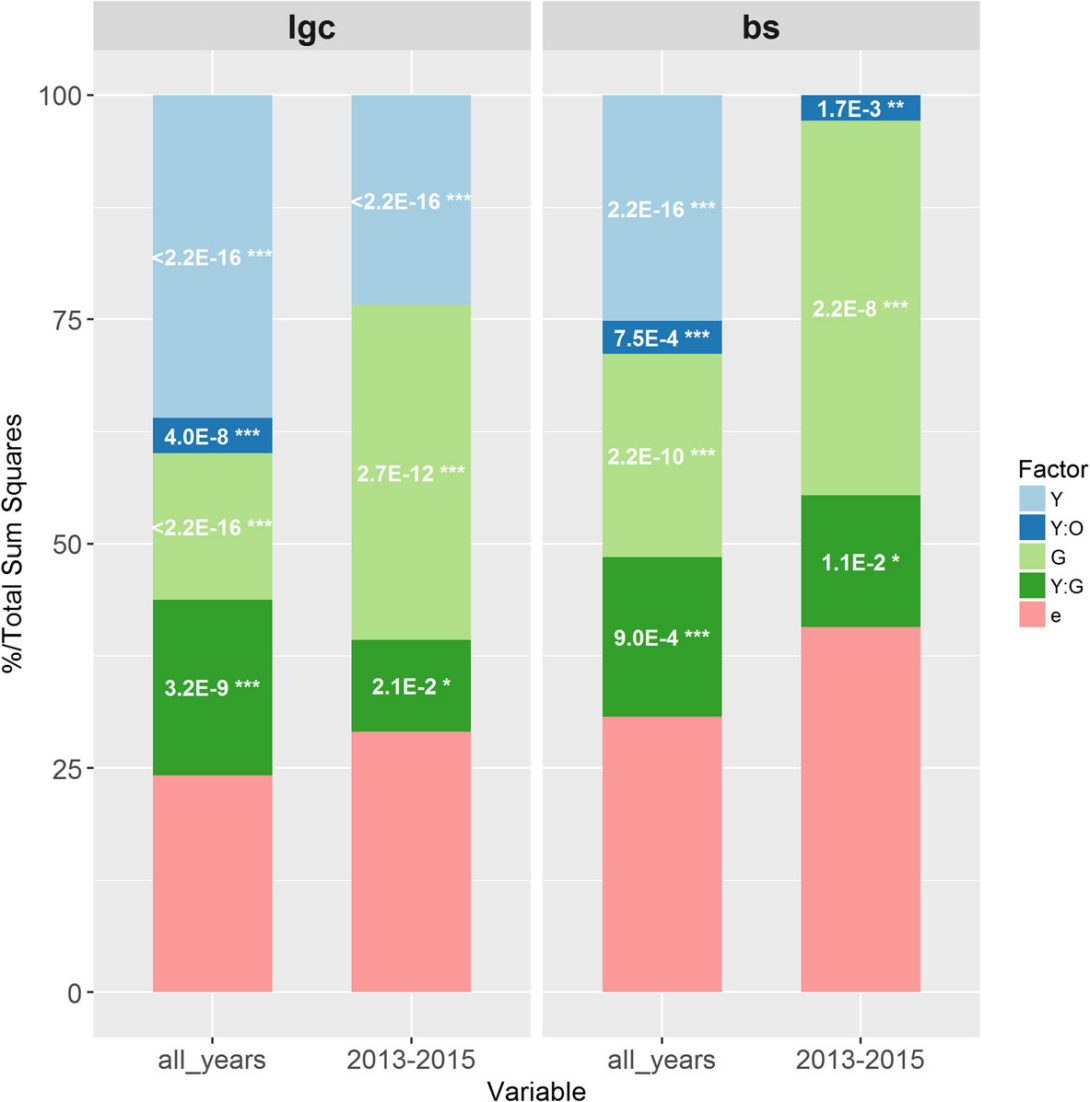
Phenotypic variance decomposition for *lgc* and *bs* considering all years and 2013 & 2015 data. For *lgc* and *bs* phenotypes, the chart displays the proportion of each fixed effect in the total sum of squares: year (Y), light blue; operator nested in year (Y:O), dark blue; genetic (G), light green; interaction year x genetic (Y:G), dark green; the random residuals, pink. *P*-values and their level of significance are indicated as following: ***P < 0.001, **0.001 ≤ *P* < 0.01, *0.01 ≤ *P* < 0.05

Considering all years, part of the total phenotypic variation was attributed in order of significance to year (*p* < 2.2E-16, 36% *lgc* and 25% *bs*), genotype (*lgc* *p* < 2.2E-16, 16% and *bs* p < 2.2E-10, 23%), genotype x year interaction (*lgc p* < 3.2E-09, 20% and *bs p* < 9.0E-04,18%) and operator (nested in year) effects (*lgc p* < 4.0E-08, 4% and *bs p* < 1.7E-03, 4%) (Fig. 1).

The *G* × *Y* interaction term for *bs* was mainly due to scale-change (73% of the sum of squares) than cross-over interactions (method 1 from [56]). By contrast, for *lgc*, the sum of squares partitioning indicated a more equilibrated contribution from each type of interaction (58 and 42% for cross-over and scale-change, respectively). Cross-over interactions were most likely due to the re-ranking of individuals with non-extreme behaviors especially in 2014 and 2016. These 2 years were characterized as non-favorable for disease development compared to 2013 and 2015: ANOVA (analysis of variance) fixed effects for *lgc/bs* equaled 2.56/NA (2014) and 2.97/0.72 cm (2016), respectively compared to 3.90/5.62 (2013) and 7.94/4.87 cm (2015). Winters from 2014 and 2016 were characterized by relatively warmer temperatures especially in February. This could be shown in terms of degree days over the winters of 2013 to 2016 (see Additional file 1).

When only the favorable years 2013 and 2015 were considered, a greater part of the total phenotypic variation for both phenotypes was attributed to the genotype: 33% for *lgc* and 42% for *bs* (Fig. 1). Heterogeneity of genetic variances was confirmed among years: only two of the 4 years (2013 and 2015) had higher significant variances due to favorable environmental effects. Therefore, we decided to keep only 2013 and 2015 for further analyses in the multi-year model, in order to capture the largest phenotypic variance due to the genetic effect.

Linear mixed models resulted in the selection of an optimal model (based on minimization of AIC (Akaike Information Criterion, see Methods) for which, in addition to the heterogeneity of genetic variances, residual variance was either common (*lgc*) or specific (*bs*) to the year. Genetic variation displayed a quantitative distribution and broad-sense heritability *H*^2^ per trait depended therefore on yearly-winter expression (Additional file 2A & B).

For the year with maximum genetic variance (2015), *H*^2^ reached 59% for *lgc* and 78% for *bs* (in comparison, for 2013 *H*^2^_*lgc*_ = 35% and *H*^2^_*bs*_ = 53%).

Significant phenotypic correlations between 2013 and 2015 (*lgc*: *r* = 0.97 ± 0.32, *p* = 8.90E-03** – *bs*: *r* = 0.54 ± 0.21, *p* = 2.91E-02*) were obtained from the corh (correlation) vcov (variance-covariance) structure. *Lgc* and *bs G* BLUPs were also highly correlated (Pearson correlation *r* = 0.57 ± 0.10, *p* = 1.48E-07*** corrected with a FDR (false discovery rate) α = 5% for multiple-testing) (Additional file 2C).

### Population stratification analysis

From the subset of 21,942 independent SNPs, population structure analysis revealed a minimization of the cross-validation error for k = 2 and k = 3 scenarii (Additional file 3A). In parallel, performing a PCA (principal component analysis) on the same subset of markers identified three components that explained 35.23% of the genetic variation in the collection with the first component explaining 27.55% of the variation (Additional file 3B). According to the ancestral fractions obtained with Admixture, the k = 3 distribution underlined three homogeneous pools of accessions belonging to Continental Europe (12%), Irano-Caucasian-Mediterranean Basin (7%) and Central-Eastern Asia (3%) groups with all remaining accessions (78%) being admixed (Additional file 3C). No obvious relation between ancestral population assignment and susceptibility to the disease was observed aside from the slight proximity of covariate Q2 (the fraction of ancestry linked to Europe Continental group) with the susceptible phenotypes (Additional file 4).

### LD decay in the GWAS panel

Pairwise mean LD estimates (*r*^2^) within each chromosome revealed a low level of dependence between markers. The range varied from 2.88E-02 ± 5.08E-02 (chromosome 8) to 4.13E-02 ± 7.71E-02 (chromosome 5) with a decrease of 20% (chromosome 5) to 31% (chromosome 4) when correcting for population structure and individual relatedness (for ${r}_{vs}^2$ average values see Methods, Additional file 5). Moreover the intra-chromosomal LD in our population set decayed very quickly within 100 to 200 bp depending on the chromosome with no significant contribution of population stratification.

### Association mapping and local LD patterns

For all *G* and *G* × *Y* BLUPs extracted from the multi-year model, QQ-plots (quantile-quantile-plots) revealed that the non-corrected ‘glm’ (generalize linear model) did not markedly inflate *p*-values (Additional file 6). Controlling for Q yielded to a slight improvement in reduction of false-positive signals (type I error) while the K model (emmax K, see Methods) allowed the best prediction of p-values with a very limited deviation from the expected p-values resulting from the null model. However, for some BLUP phenotypes (*lgc* and *bs* (*G* ***×*** *Y*)_2015_), whatever the correction applied, both naïve ‘glm’ model, K and Q models led to equivalent estimations of p-values. Interestingly, controlling for both relatedness (K) and structure (Q) did not provide better results than the K model (Additional file 6).

The multi-locus mixed model approach (correcting for relatedness) detected 8 associations for all *G* and *G* × *Y* BLUP phenotypes with one colocalization for *lgc G*, (*G* ***×*** *Y*)_2013_ and (*G* ***×*** *Y*)_2015_ BLUPs and PVE (percentage of variance explained) ranging from 17.1 to 45.5% (Table 1). Two main signals on the global *G* terms were localized on chromosomes 5 (LG5_5394803) and 6 (LG6_15273858), and associated respectively with *bs G* BLUP (*p* = 8.10E-06) and *lgc G* BLUP (*p* = 1.2E-07) with PVE reaching 25.9 and 41.4%, respectively. The colocalization between *G* and *G* × *Y* BLUPs on *lgc* indicated a modulation according to the year of both allelic effect (α_2013_ = 0.14, α_2015_ = 0.26, ln(x + 1) scale on the length in cm) and PVE on the phenotype (PVE_2013_ = 40.1%, PVE_2015_ = 45.5%).

**Table 1:** Detailed information about the 11 detected associations using *G* and *G* × *Y* BLUPs for *lgc* and *bs*

SNP ID^a^	Maj/Min Allele^b^	Chr^c^	MAF^d^	Phenotype	GWAS model	P-val^e^	P-val_reg_^f^	P-val _FDR_^g^	α^h^	PVE^i^	SNP location	Gene ID *P. persica* *v1.0* (*v2.1*)	TAIR10 BLASTp homology^j^	
													*Description*	*E-value*
LG6_15273858	A/G	6	0.43	lgc *G*	multi-locus	1.22E-07	1.22E-07	–	0.20	41	Intron	*ppa018341m.g*	AT5G41980 Unknown Protein CONTAINS Harbinger transposase-derived nuclease domain	7E-51
					GEMMA	2.88E-07	–	2.21E-03	0.21	–				
				bs *G*					0.28	–				
			0.42	lgc (*G* ***×*** *Y*)_2013_	multi-locus	3.85E-07	3.85E-07	–	0.14	40				
			0.43	lgc (*G* ***×*** *Y*)_2015_	multi-locus	1.04E-06	1.04E-06		0.26	46				
LG5_5394803	G/A	5	0.19	lgc *G*	GEMMA	9.46E-06	–	4.88E-02	−0.15	–	Intron	*ppa000004m.g* (Prupe.5G050700)	AT4G17140 Predicted: Pleckstrin (PH) domain containing protein	0.0
				bs *G*					−0.38					
					multi-locus	8.10E-06	1.05E-08	–	−0.32	26				
LG3_10897844	T/A	3	0.32	bs *G*	multi-locus	4.60E-03	2.73E-06	–	−0.31	19	CDS*	*ppa017602m.g* (Prupe.3G152500)	AT5G17460 Glutamyl-tRNA (Gln) amidotransferase subunit C	2E-66
LG2_22535054	T/C	2	0.30		multi-locus	8.08E-05	2.27E-06		−0.29	17	Intron	*ppa023961m.g* (Prupe.2G242100)	AT3G46850 Subtilisin-like protease SBT4.6	0.0
LG3_4322444	G/A	3	0.39	bs (*G* ***×*** *Y*)_2015_	multi-locus	7.22E-06	6.98E-06		0.46	43	CDS*	*ppa008956m.g* (Prupe.3G072200)	AT1G52360 Coatomer subunit beta’-2	6.8e-117
LG5_4842835	T/A	5	0.19		multi-locus	7.00E-05	1.68E-05		−0.25	20	Intron	*ppa020388m.g* (Prupe.5G044100)	AT4G18010 Type I inositol polyphosphate 5-phosphatase 2, IP5P2	0.0
LG7_18047191	C/T	7	0.45	lgc *G*	GEMMA	4.47E-07	–	2.72E-03	−0.05	3	Intron	*ppa001722m.g* (Prupe.7G179600)	AT5G66030 GRIP Protein	0.0
				bs *G*					0.19	12				

CDS* = Coding Sequence with SNP polymorphism impacting a non-synonymous substitution

*ppa018341m.g is not referenced in P.persica v2*

^a^SNP ID noted as LG[chromosome number]_[physical position in bp]

^b^Major/Minor Allele

^c^Chromosome

^d^Minor Allele Frequency

^e^SNP *p*-value (considered as the only fixed regressor in the model)

^f^SNP *p*-value from the multi-locus optimal-model (multiple regressors)

^g^SNP *p*-value in mvLMM corrected for FDR (False Discovery Rate)

^h^Allelic effect (ln(x + 1) scale)

^i^Percentage of phenotypic variance explained by the SNP

^j^Functional annotation obtained using BLASTp homology on *A.thaliana* (TAIR10 database)

Additional associations were noticed on *bs* (*G* ***×*** *Y*)_2015_ BLUP compared to *G* BLUP with loci detected on chromosomes 3 (LG3_4322444, *p* = 7.22E-06) and 5 (LG5_4842835, *p* = 7.00E-05). The number of associated loci per phenotype ranged from one (*lgc G*, (*G* ***×*** *Y*)_2013_ and (*G* ***×*** *Y*)_2015_ BLUPs) to three (*bs G* BLUP) with an important contribution of the additive genetic variance to the total phenotype variability (*h*_*lgc G*_^2^=0.41, *h*_*bs G*_^2^=0.62). Despite the high phenotypic correlation (*r* = 0.57 ± 0.10***, Pearson correlation) between the two phenotypes, no marker colocalization was found.

Furthermore, 3 SNPs impacting both *lgc G* and *bs G* BLUPs were identified on chromosomes 5, 6 and 7 with the mvLMM (multi variate linear mixed model) (Table 1). Genetic correlation *r*_*g* (*lgc* − *bs*)_ reached 0.52 (MLE: maximum likelihood estimate) suggesting a common genetic pattern underlying *lgc* and *bs* variabilities. Among these markers, 2 SNPs were previously detected with the multi-locus mixed model for either *lgc* (LG6_15273858, p_FDR_ = 2.21E-03) or *bs* (LG5_5394803, p_FDR_ = 4.88E-02) with similar allelic effects. The association detected on chromosome 7 using mvLMM, accounted for a lower but significant part of the total phenotypic variability with PVE = 3.0% for *lgc* and PVE = 12.0% for *bs.*

**Fig. 2:**
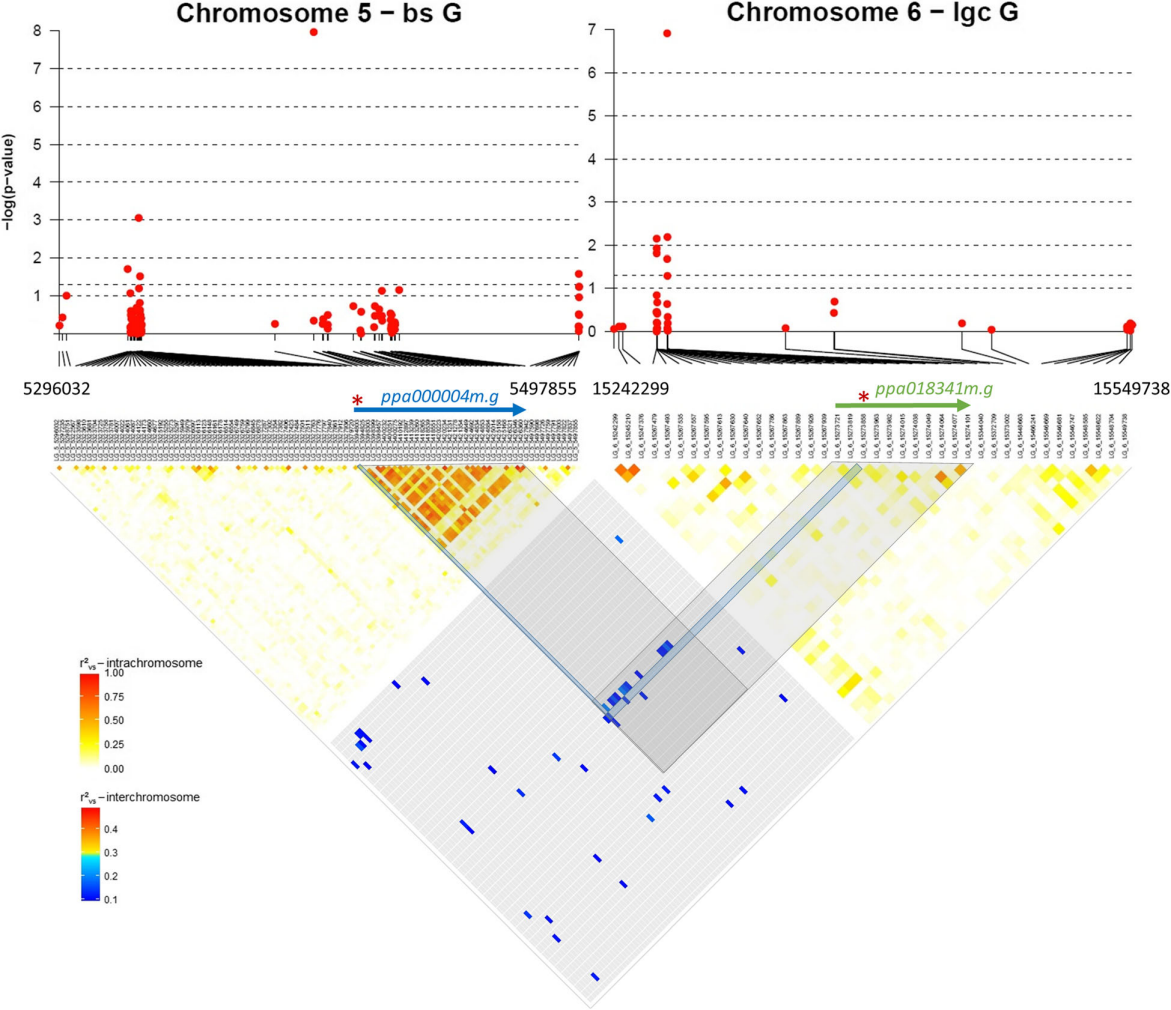
Manhattan plots of the –log10(*p*-values) over physical positions in windows surrounding LG6_15273858 and LG5_5394803 associations. Genomic windows are approximately 201.82 kb (chromosome 5) and 307.44 kb (chromosome 6). Significance level was determined with eBIC criterion. As an indication, Bonferroni threshold = *−log10*(p-value_thr_) = 6.10. Detected SNP are shown with red stars and overlapping candidate genes indicated with arrows. Heatmaps of pairwise LD estimates (${r}_{vs}^2\Big)$ were drawn both within the genomic window around each candidate and between the two genomic frames (inter-chromosome scale). Different colors and scales are used to represent the pairwise LD estimates between inter or intra SNP pairs. Only inter-chromosomal pairwise LD ${r}_{vs}^2$over the 99th percentile of the ${r}_{vs}^2$ distribution are displayed

Manhattan plots displaying *p*-values for the main loci LG6_15273858 and LG5_5394803 in multi-locus and multi-variate mixed models are shown in Fig. 2. Intra-chromosomal LD of the two chromosomes have different patterns around each detected locus (Fig. 2). In the genomic region around LG5_5394803, a relatively high LD block overlapped precisely with *pp0000 4 m.g*, the underlying candidate gene (cf Table 1). This might be related to the above-mentioned higher mean *r*^2^ of chromosome 5 compared to all other chromosomes (Additional file 5). In contrast, SNP peak LG6_15273858 and all other candidate loci (see Additional file 7) appeared to be located in a low LD region with no specific genomic structure.

**Fig. 3:**
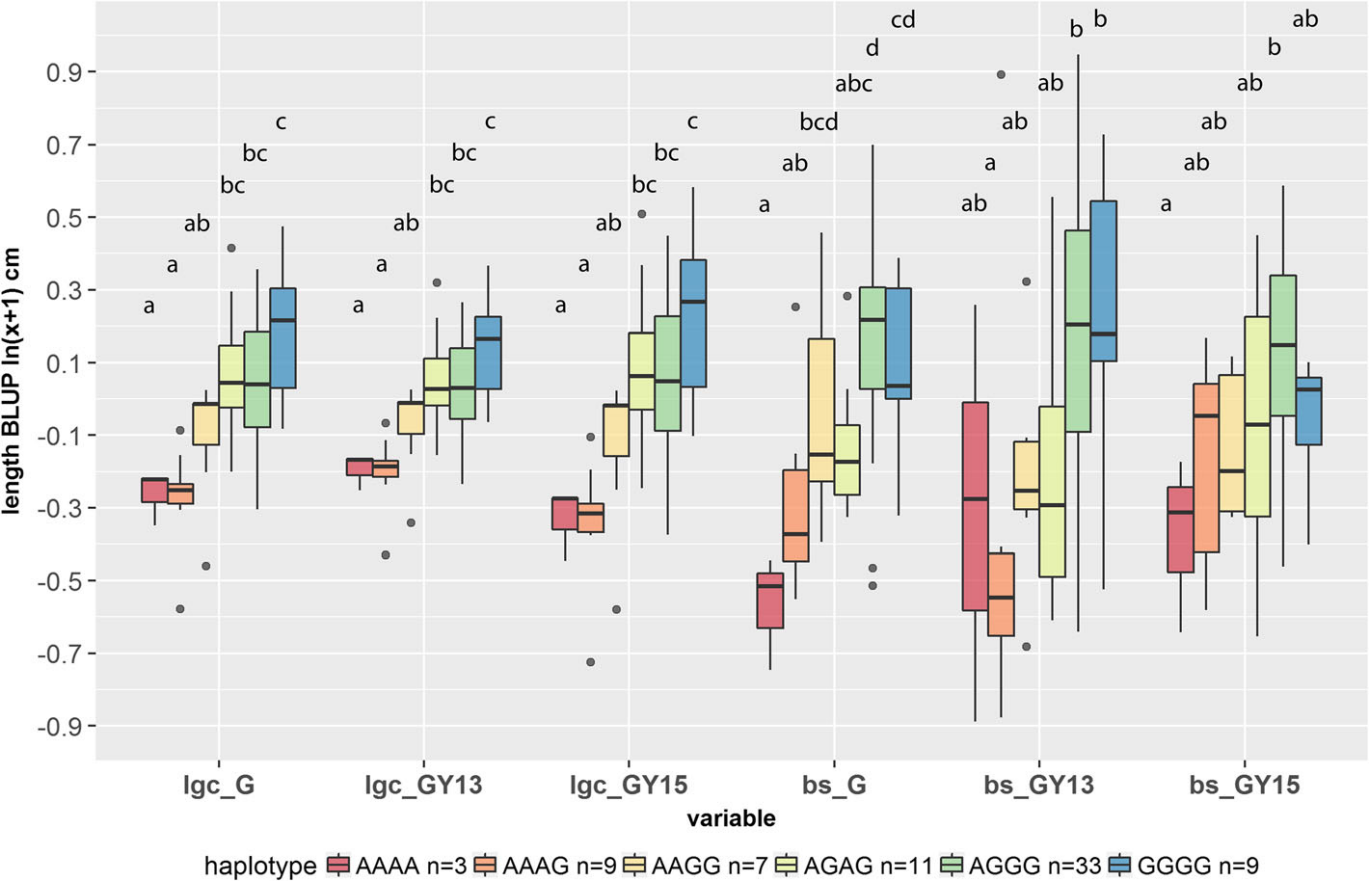
*G* and *G* × *Y* BLUP distributions according to the haplotype constituted by LG6_15273858 and LG5_5394803. Statistical differences between groups are indicated – HSD post-hoc tests on each *G* & *G* × *Y* term. For *bs G* and (*G* ***×*** *Y*)_2015_ BLUPs, tests were performed on residuals from an ANOVA model accounting the other associated loci from multi-locus model (LG3_10897844 and LG52_22535054 for *bs G* LG3_4322444 and LG5_4842835 for *bs* (*G* ***×*** *Y*)_2015_). Y-axis values are indicated in ln(x + 1) scale. Haplotype class ‘AGAA’ (1 individual) was discarded in the boxplot

As these two main SNPs were detected by the two mapping approaches, we focused on the effect of the haplotype, constituted by the genotypes on LG6_15273858 and LG5_5394803, on the total phenotypic variance of both *lgc* and *bs*. Both loci seemed to demonstrate an additive relation between allelic dose and the phenotype considering either *G* or *G* × *Y* BLUPs (Fig. 3) with no epistasis which was confirmed by performing an ANOVA (non-significant SNPxSNP interaction term *p* > 0.05). Individual effects of LG6_15273858, LG5_5394803 and all other detected loci on their respective *G* or *G* × *Y* BLUP phenotypes are shown in Additional file 7.

Interestingly, a relatively high LD square correlation value (${r}_{vs}^2$=9.76E-02), higher than the 99th percentile of ${r}_{vs}^2$ inter-chromosomal distribution, was revealed more specifically between the two main candidates LG6_15273858 and LG5_5394803 (Additional file 8). This correlation between associated loci proved to be within a longer LD block (Fig. 2) revealing an inter-chromosomal linkage between the underlying candidate genes *pp00000 4 m.g* (chromosome 5) and *pp01834 1 m.g* (chromosome 6). Likewise, the correlation between LG5_5394803 and LG5_4842835 (${r}_{vs}^2$=2.94E-01) detected both on chromosome 5 appeared to be significantly higher than the 99th percentile of ${r}_{vs}^2$ intra-chromosomal distribution (Additional file 8).

The genotype group distribution from all detected SNPs from the multi-year model BLUPs was tested on the within-year model BLUPs with heterogeneous significance levels according to the year and the marker PVE. Marker LG7_18047191 was only significant for *bs2013* and *bs2015*. Significance of the two main loci was confirmed individually for *lgc2013*, *lgc2015, lgc2016* and *bs2015* with LG6_15273858 and for *bs2013* and *bs2015* considering LG5_5394803 (uni and multi-factorial ANOVA and post-hoc tests in Additional file 9). Strict-sense heritability estimates ranged from 10.85% (*lgc2014*) to 64.46% (*bs2015*) with non-negligible values even in the non-favorable years 2014 and 2016. Only *bs2016* led to non-significant results for all detected markers (Additional file 9).

### Haplotypes associated with partial resistance

We found three groups of haplotypes among the core-collection having several SNPs associated to canker resistance; 18, 45 and 9 haplotypes shared 3, 5 and 2 markers associated to *lgc G*, *bs G* and *bs* (*G* ***×*** *Y*)_2015_ BLUPs, respectively (Additional file 10). Mean comparison was performed on BLUP values from multi-year and within-year models after discarding low frequency classes in order to limit false-positive findings (type I error). Five haplotypes were identified with favorable alleles and stability of the resistance through years (1 with *lgc G*, 3 with *bs G*, and 1 with *bs* (*G* ***×*** *Y*)_2015_ BLUP, see Additional file 10). Accessions A1814 and Harlayne showed two and all of the three defined favorable haplotypes, respectively. A0008 and A1314 were in the same way noticeable for their partial resistance (see Additional file 2C) but their corresponding haplotypes were either not located in the most resistant groups or, if they were, they appeared to be unstable between years (see Additional file 10).

The mean comparison test was non-significant for the within-year model *lgc2015* BLUP for the first group of haplotypes (related to the 3 candidate loci associated to *lgc G* BLUP) which might be due to a loss of power when haplotype number (18) is high compared to population size (ANOVA test *p* = 0.1322). None of the comparison tests between haplotypes were significant for years 2014 and 2016, which might be explained by the low genetic variance in these years where there was low disease severity.

### Candidate genes for resistance to bacterial canker

Putative candidate gene annotations for all multi-locus and mvLMM associated SNPs were reported in Table 1. To date, none of the associated SNPs were reported for disease resistance in *a Rosaceae* species or colocalized with previously known QTL. It is noteworthy that, over all the 7 detected loci, only 2 candidates LG3_10897844 and LG3_4322444 targeted polymorphisms in coding sequences (CDS) of *ppa017602m.g* and *ppa008956m.g*, respectively. Mutations were both non-synonymous with one substitution (K49 M in *ppa017602m.g*) leading to a deleterious predictive effect on the corresponding protein, a glutamyl-tRNA (Gln) amidotransferase subunit C protein. All other loci comprising the two most significant loci LG6_15273858 (*ppa018341m.g*) and LG5_5394803 (*ppa000004m.g*) were localized in introns. The associated functional annotations of the two main candidate genes were related to Harbinger transposase-derived nuclease and Pleckstrin homology-like (PH) domains, corresponding to a superfamily of transposons found in plants and animals [57] and a conserved domain particularly abundant in proteins involved in signal transduction pathways [58], respectively.

## Discussion

We aimed at investigating the genetic pattern of partial resistance of apricot to bacterial canker caused by *P. syringae* using a broad genetic basis in the species. We showed that, although being polygenic, bacterial canker partial resistance appeared to rest upon a limited number of additive components: 5 SNPs on chromosomes 2,3,5,6 and 7 partially explaining 45 and 62% of the total phenotypic variance of canker (*lgc*) and superficial browning (*bs*) lengths, respectively. A large part of the genotypic variance was due to a winter effect allowing the detection of two additional loci on chromosomes 3 and 5 in the year most favorable for the expression of canker symptoms. Among the 7 candidate loci, two main SNPs on chromosome 5 and 6 displayed additive contributions reaching 41 and 26% of the variation of *lgc* and *bs*. Furthermore, these two markers showed a long-range inter-chromosomal LD revealing a multi-locus-linked selection event through population history. Due to the short decay of LD in apricot, candidate loci were identified with a high resolution leading to the identification of promising candidate genes involving a potential signaling cross-talk between abscisic (ABA), jasmonic (JA), and salicylic (SA) acids, molecules known to mediate long-distance signaling in plant-pathogen interactions. Overall, this study contributed to the very first characterization of the genetic variation and genomic determinants of partial resistance to bacterial canker in a fruit tree species.

### Detection of genomic regions controlling variation of partial resistance to bacterial canker is dependent on winter frost intensity

As highlighted by the heterogeneity of genetic variances across 4 years, genetic variation was highly dependent on winter-frost severity with low genetic variations in 2014 and 2016 while the highest were registered in 2013 and 2015. Considering this environmental dependency, the screening and characterization of cultivars for bacterial canker resistance was a difficult task. Other limits of bacterial canker phenotyping concern time-consuming efforts in the orchard, a very long period of symptom development, the need to calibrate the observations between operators and no guarantee of getting significant genetic variation between individuals. Marker-assisted selection (MAS) programs would be in that case an attractive, powerful and cost-efficient alternative to phenotyping.

Among the two most severe years, broad-sense heritability estimates ranged from 35% (*lgc* in 2013) to 78% (*bs* in 2015) showing a considerable level of variation of susceptibility in the core-collection. Previous studies on other patho-systems in fruit and forest trees have reported moderate to high heritability estimates ranging from 30 to 40% for pitch canker resistance in loblolly pine [59], from 70 to 89% in peach x *Prunus davidiana* [60] and 60 to 87% in apple [61] for powdery mildew resistance. Importantly, these studies reported the crucial need for controlling GxE interactions in order to estimate accurate genetic effects in the face of environmental noise in pluri-annual data.

The two phenotypes *lgc* and *bs* displayed a high correlation (Pearson correlation *r* = 0.57 ± 0.10, *p* = 1.48E-07***). Regarding the important contribution of the genetic factor to the total variance of the phenotypes, this correlation suggests that a common genetic pattern may control both phenotypes.

We identified a total of 11 associations, over 7 candidate SNPs on chromosomes 2, 3, 5, 6 and 7, linked to the variance of *lgc* and *bs G* and *G* ***×*** *Y* BLUPs. Among the stable associations, two main SNPs were detected on chromosomes 5 (LG5_5394803) and 6 (LG6_15273858) and explained 41 and 26% of the variation of *lgc* and *bs,* respectively*.* In the case of *lgc*, a unique locus (LG6_15273858) was detected both for *G* ***×*** *Y* interaction BLUPs and the overall *G* term with a modulation of the allelic effect and the PVE according to the year due to the heterogeneity of winter temperatures. Moreover, new associations for *bs* were detected in 2015 which was the most severe year in terms of disease expression, suggesting that data on specific years and climatic conditions have the potential to reveal additional regions linked to disease resistance. Similar results have been obtained in the case of QTL mapping on tree architecture [62] and phenological traits [63] in apple underlying the usefulness of focusing on *G* and *G* ***×*** *Y* terms to target both stable and environment-specific genetic determinants.

### Candidate genes can be found by exploiting the quick LD decay in apricot

Mapping resolution in GWAS mostly depends on the decay of LD: the more the LD decreases, the sharper the resolution appears around the detected locus [64]. Considering the original reproductive characteristics of the family, most *Rosaceae* species are cross-pollinating because of their self-incompatibly system. This results in a high number of effective recombination events and a rapid expected LD decay over the genome [65]. In our study, global mean LD over all chromosomes decayed over very short distances (100 to 200 bp) considering both corrections for population stratification and uncorrected estimates. Moreover, LD in the case of our population is not likely to rely on confounding structure effects related to differences in allele frequencies between groups resulting from non-random mating [66]. These results are congruent with those reported in the previous association study on apricot [30] based on a population comprising the major part of the material used in our work.

Regarding all these observations, GWAS is a very suitable approach to map the genetic regions linked to bacterial canker partial resistance in our diversity panel with a very precise resolution.

To date, none of the putative candidate genes or domains associated with the SNPs identified here have been reported for plant resistance function in the *Rosaceae* family. The two main SNPs LG6_15273858 and LG5_5394803 were located in genes with significant homologies to Harbinger transposase-derived nuclease (*ppa018341m.g*) and Pleckstrin Homology PH (*ppa000004m.g*) domains, respectively. These motifs are conserved domains found in a wide range of uncharacterized proteins with functions respectively in, chromatin remodeling [67] and signal transduction through interaction with membrane phosphoinositides [68, 69]. A role for phosphoinositides (binding to pleckstrin homology domain - *ppa000004m.g*) in regulating plant nuclear functions and possibly transcriptional activities through chromatin remodeling (Harbinger transposase-derived nuclease - *ppa018341m.g*) was revealed as a common response to a large range of both abiotic and biotic stresses [70]. It is noteworthy that the protein encoded by the candidate gene *ppa023961m.g* located on chromosome 2 (SNP: LG2_22535054) belongs to a large family of proteases: the subtilisin-like proteases (or subtilases) whose involvement in plant defense responses has been more and more described recently [71]. Two closely related members of the P69 subtilase family (*P69B* and *P69C*) were shown to be transcriptionally activated after *Ps* DC3000 infection with an elicitation from SA and JA [72, 73]. Although the link between *SBT4.6* encoded by *ppa023961m.g* and plant resistance remains to be investigated, transcripts from a close relative *SBT4.14* (also known as *AtXSP1*) were evidenced as contributing to xylem differentiation in *A. thaliana* [74]. Moreover, SBT4.6 subtilase was predicted to be in the extracellular space (Uniprot database) suggesting a role in recognition of *Ps* before entry and colonization through the xylem, in the case of a compatible interaction. Interestingly, the G protein β WD-40 repeat, one of the domains of the protein encoded by another candidate gene (*ppa008956m.g* for *bs* (*G* ***×*** *Y*)_2015_ association), had been previously shown to bind in-vitro with PH domains (*ppa000004m.g*) [68]. It has been shown in *A. thaliana* that myo-inositol polyphosphate 5-phosphatases, a large family englobing IP5P2 (*ppa020388m.g* including detected loci LG5_4842835), hydrolyze a wide range of phosphoinositide phosphate substrates and is involved in stress responses through the abscisic acid (ABA) signaling pathway [75]. In addition, research that deployed transcriptional approaches demonstrated that the ABA pathway was one of the main targets of effectors secreted by *Ps* [76]. Regarding all their functions, most of the candidate genes or domains seem to support a role in a regulatory network involving signal transduction through membrane phosphoinositides in a cross talk involving ABA, with SA and JA, potentially. ABA mostly acts as negative regulator of disease resistance with an antagonistic effect on SA and JA [77, 78].

In addition, over all the seven candidate loci, only two single polymorphisms targeted CDS with non-synonymous substitutions. This observation emphasizes the importance of introns which can affect gene expression level or induce alternate splicing with an impact on the phenotype. Moreover, the genotype data we used in the association mapping was restricted to variants located in gene-space regions. In turn, we may have missed inter-genic allelic diversity and regulatory variants potentially contributing to phenotype variation. The role of non-coding DNA such as promoters or/and enhancers in the variability of susceptibility to plant disease resistance has been demonstrated in several patho-systems including rice/*Xanthomonas oryzae* pv. *oryzae* [79] and maize/maize rough dwarf virus [80] and would be worth further investigation in the case of bacterial canker partial resistance.

These results open the door for subsequent validations to confirm the candidate loci polymorphisms over a pool of genetic resources and the correlations between the SNP genotype and the susceptibility level to the disease. To achieve this goal, pyro-sequencing could be performed in order to confirm the haplotypes. Moreover, in order to take into account the significance of non-coding polymorphism candidates from our study, validation of the candidate genes could be performed with qRT-PCR across a pool of favorable and unfavorable haplotypes identified in our study.

### Combining genome-wide multi-locus and multi-variate association models is a powerful method to decipher the genomic pattern of partial resistance to bacterial canker

There have been very few association studies using both genome-wide multi-locus and multi-variate mixed models [81, 82]. Our approach combining both methods proved to be powerful in limiting the number of candidates by controlling for LD, capturing a large part of broad-sense heritability and detecting common genetic variants impacting the two phenotypes.

The two main SNPs LG5_5394803 and LG6_15273858 were associated independently with multi-locus models on *lgc* (chromosome 6) and *bs* (chromosome 5) and co-detected on both phenotypes with the multi-variate model. The higher correlation between LG5_5394803 and LG6_15273858 (${r}_{vs}^2$=9.76E-02) compared to background pairwise loci could explain the lack of co-localization with the multi-locus model. Indeed, the multi-locus model corrects for potential spurious associations due to intra or inter-chromosomic LD by re-estimating genetic variance at the stepwise inclusion of each associated locus as regressor in the model [50]. The drawback of such correction is that pleiotropic genetic variants cannot be detected, which in turn emphasizes the advantage of using multi-variate association models as a complementary approach. Noteworthy, multi-variate mixed models, by taking advantage of correlations between phenotypes can potentially also capture variants with smaller effects than those detected with traditional uni-variate analyses [83]. As an example, this allowed us to detect the small, but significant effect of LG7_18047191 (chromosome 7) on both *lgc* and *bs G* BLUP (with PVE reaching 3 and 12%, respectively).

Despite the small size of our core-collection (73 accessions), we have successfully shown that our method has enough power to detect marker-trait associations for bacterial canker resistance. Nevertheless, our population might not have been large enough to capture SNPs with minor effects due to a lack of statistical power with type II (false-negative) errors. An interesting next analytic perspective would be the use of genomic selection (GS) models on a higher number of individuals [84]. This would assess the effect of all markers and in particular low-effect variants in the phenotypic variance regardless of their frequency in the population, allowing to validate the candidates while detecting new loci and thus giving an overall better estimation of the genetic effects [85]. In our case, due to genotypic sampling bias, these genetic effects could have been either overestimated for the detected loci [86] or underestimated for rare variants possibly linked to resistance. For instance, the cultivars A0008 and A1314 noticed for particular phenotype of partial resistance were not highlighted by the analysis of haplotypes in our study, but they might be relevant material to cross in progenies. GS models or linkage analysis using bi-parental, multi-parental or interconnected progenies [63, 87–89] derived from this material could thus map additional rare variants which could have been removed in our study (filter MAF < 0.05).

### Specific intra and inter-chromosomal LD patterns give insight into genomic variation through population history

This study allowed us to reveal intra and inter-chromosomal patterns with either low or high LD structure around the candidate loci. More specifically, one of the main detected SNPs on chromosome 5 (LG5_5394803) displayed a high LD block overlapping precisely with the underlying gene *ppa000004m.g,* while all other associations targeted low LD regions even considering the other detected loci (LG5_4842835) located 552 kb downstream. We also detected a high level of inter-chromosomal LD (${r}_{vs}^2$=9.76E-02) between the two main detected SNPs included in the candidate genes of interest *ppa000004m.g* and *ppa018341m.g* on chromosomes 5 and 6.

A high level of LD may often be interpreted as a direct effect of selective breeding in a genomic area where genes of agronomic interest are gathered. It also could be possibly due to a population structure confounding effect but we have shown for both intra and inter-chromosomal LD patterns that high levels of LD remain after correcting for population structure and relatedness, indicating a biological relevance behind these allelic associations. Considering the broadly diverse germplasm we used through this study, resulting from numerous recombination episodes through evolution, such strong LD patterns, especially between unlinked loci (over different chromosomes) are unexpected. These observations could then suggest a possible linkage due to a strong multi-locus linked selection acting as an evolutionary counterforce preserving an efficient and functional synergy between *ppa000004m.g* and *ppa018341m.g*. The same hypothesis could be drawn from the long-range LD between LG5_5394803 and LG5_4842835 impacting either *G* or *G* ***×*** *Y* variation of superficial browning.

High LD between physically unlinked loci is likely to happen when strong selection is applied in conjunction with epistasis, pleiotropy [90] and/or co-adaptation on the basis of biological mechanisms leading to a “functional linkage” as it has been described in *Arabidopsis thaliana* with the genes couple GS-OH and MAM1 [91]. These two genes located respectively on chromosomes 2 and 5, showed a significant long-range genome-wide correlation and epistasis effects with favorable allelic combinations impacting the biosynthetic pathway related to herbivore resistance [91]. While the biological relation between *ppa000004m.g* and *ppa018341m.g* needs further investigation, LG5_5394803 and LG5_4842835 were included in two genes (encoding PH domain protein and IP5P2) related to phosphoinositide substrates and membrane components. The proximity of ontologies between these two genes located 0.5 Mb apart suggests a functional linkage.

Both long-range and inter-chromosomal LD between some of the markers of interest and the annotation of their underlying genes emphasize the idea of a functional network involved in bacterial canker resistance. In addition, the particular LD block structure of *ppa000004m.g* could be explained by a prevalent role of this gene in this network, possibly as an immediate-early gene involved in the initial steps of *Ps* infection. The function of *ppa000004m.g* interacting with membrane phosphoinositides corroborates the key-gene hypothesis as the plasma membrane is one of the first battle grounds in the plant-pathogen interaction.

## Conclusion

We aimed at targeting the genetic pattern controlling susceptibility to bacterial canker in apricot by exploring the natural genetic diversity in a panel of 73 apricot accessions. To achieve this goal, we used a very dense set of 63,236 SNPs spread over the genome and performed association mapping using both multi-locus and multi-variate mixed models on two correlated phenotypes resulting from controlled inoculations in the orchard during 2 years favorable for disease expression.

A total of 11 associations was detected with two main components located on chromosomes 5 and 6 contributing to a large part of additive variation of the phenotype (41 and 26% of PVE). Putative candidate genes linked to the abscisic acid pathway were reported with a very precise resolution given the extremely low LD shaping the apricot tree genome organization.

In terms of methodology, we also demonstrated the complementary of genome-wide association multi-locus and multi-variate mixed models allowing i) estimation of the additive individual contribution of all associated loci in the total phenotypic variation and ii) detection of common genetic variants impacting the two phenotypes. Subsequent analyses considering a bigger GWAS population and/or the use of bi or multi-parental QTL mapping approaches would allow a further validation of the associated loci.

In a breeding context, further research is required to assess the extent of resistance due to all identified loci relative to the diversity of *Ps* in the orchard. Combining resistance genes showing major and strain-specific effects with genes displaying minor and non-specific effects was suggested to be an efficient strategy to preserve sustainability of the resistance by generating contradictory selection pressures on pathogen evolution [92, 93]. In this perspective, the loci detected in this study could be used in MAS in order to complement the global multi-trait selection strategy [65]. Gene pyramiding would be a feasible approach not only for apricot trees but also considering in a larger perspective other *Prunus* species because of the synteny between their genomes [94].

## Methods

### Plant material

A core-collection composed of 73 accessions of apricot trees was used for this study (60 accessions were previously investigated for genetic determinism of resistance to Plum Pox virus [30]). This population was grafted on Manicot apricot rootstock from 1995 to 2001 at the INRA experimental station ‘Domaine de l’Amarine’ (Gard, France). Name, geographical origin and further information of the plant material are presented in Additional file 11.

### Experimental design & phenotyping

Controlled inoculations over the core-collection (one tree for each accession, three replicates per tree) were performed annually at the end of November over 4 years (2013 to 2016) according to a previously described procedure [14]. Briefly, bacterial inoculum was prepared from a culture of strain (called ‘41A’, highly aggressive and initially isolated from an infected apricot orchard) from phylogroup 2 of *Ps* [37] grown on King’s B medium [38] for 48 h at 24 °C. The concentration of the suspension was adjusted with sterile demineralized water to 10^8^ CFU.ml^− 1^ using a spectrophotometer.

**Fig. 4:**
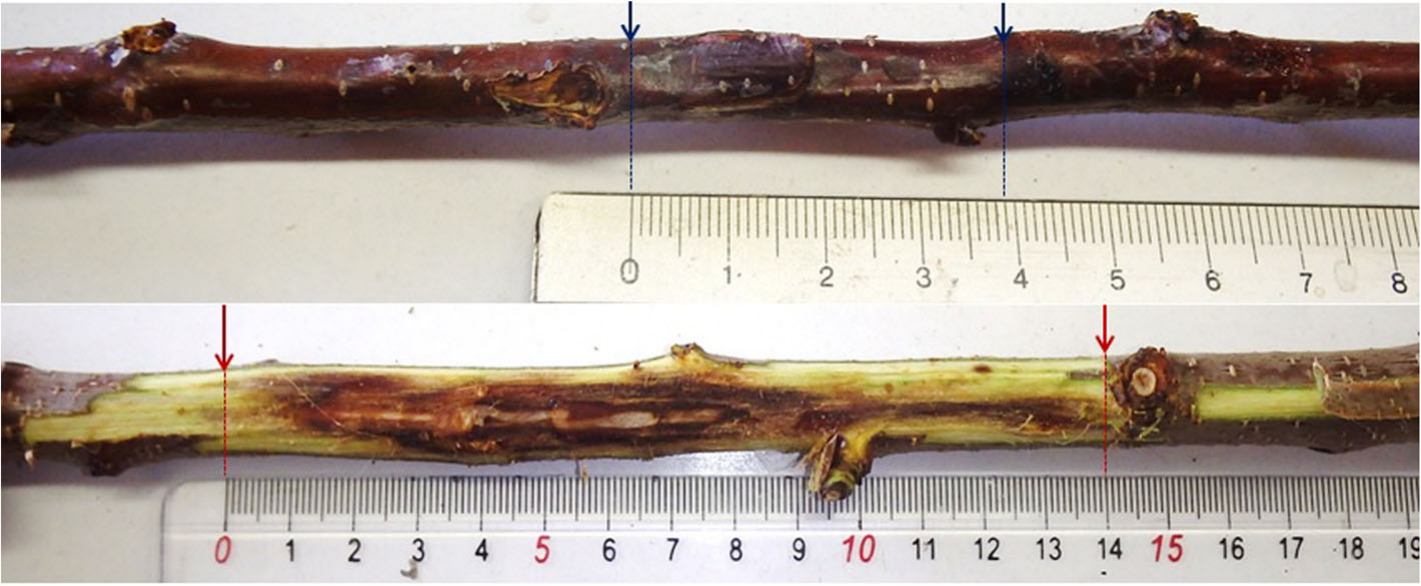
Photograph of typical bacterial canker symptoms on branches after controlled inoculation. Canker length *lgc* (blue arrows) and superficial browning *bs* (red arrows) as observed 6 months after inoculation

Three one-year old shoots per tree were injured by performing a standardized one-centimeter-long incision in the bark. A 25 μl aliquot of inoculum was injected by pipetting through the wound before wrapping with a parafilm band. For each accession, an additional shoot served as a negative control and was inoculated with the same volume of sterile water. Symptoms were allowed to develop for 6 months in the orchard before pruning of inoculated shoots for disease visual assessment. Evaluation of phenotypes was carried out by several operators according to a balanced design in order to be able to assess the effect of operators on phenotypic variability. After inoculation, and according to the pedo-climatic conditions and to the genotypes, a degradation of cambium tissues occurred. The symptoms (Fig. 4) issued from the destruction of the tissues are characterized by an alteration of the longitudinal and the lateral shapes of the shoot with a flattening zone observed and measured (the length of the longitudinal flat-zone is named “lgc”). These external symptoms can be associated with internal disorders among them superficial browning is measured as indicator of the reaction (the length of the longitudinal browning zone is named “bs”). These two indicators jointly related to the observed susceptibility to bacterial canker are used to phenotype the accessions).

Furthermore, several covariates that might have been correlated with the phenotypes were assessed, including the diameter of the lower-part of the shoot as a measure of the vigor of the accession [39] and the height of the branch in the tree as a measure related to the local temperature close to the inoculation point.

### Statistical modelling of phenotypic data

All statistical analysis were performed using R software (https://www.r-project.org/) and the ASReml-R package [40]. Prior to modelling, phenotype distribution was normalized using a ln(x + 1) transformation in order to achieve a normal distribution of the residuals (Shapiro test *p* > 0.05).

Analyses of Variance (ANOVA) were used to assess significance of fixed effects: year, operator, genotype, genotype-by-year interaction and the fixed covariates diameter and height of the branch on lgc and bs according to the following model:

$$ {P}_{ijk}=\mu +{Y}_i+{\left(Y/h\right)}_i+{\left(Y/\phi \right)}_i+{Y}_i/{O}_j+{G}_k+{G}_k\times {Y}_i+{e}_{ijk} $$

where *P*_*ijk*_ is the phenotypic value of accession *k* noted by the *j*^*th*^ operator in year *i*, *μ* the overall mean, *Y*_*i*_ the effect of year *i*, (*Y*/*h*)_*i*_ and (*Y*/*ϕ*)_*i*_ the nested effects of the shoot diameter and height, respectively within the year *i*, *Y*_*i*_/*O*_*j*_ the nested effect of the *j*^*th*^ operator within the year *i*, *G*_*k*_the effect of the accession *k*, *G*_*k*_ × *Y*_*i*_ the interaction term between year *i* and genotype *k*, and *e*_*ijk*_ the random independent and identically distributed residual term.

Two main approaches have been conducted for characterizing the influence of the inoculation into the observed accessions: a multi-years and within-year models through the use of linear mixed models.

The multi-years model integrated the effect of the Year (Y) (as fixed factor) and both – Genotype (G) and Genotype x Year (G x Y) interactions (as random factors). This model was used to model correlations of the same phenotype between years. Preliminary analysis revealed that genetic variances differed significantly between years. Thus, a homogenous correlation (corh) variance-covariance (vcov) matrix was fitted on the interaction term in the model. The residual error was modeled either for all years or for each year independently. In order to select the optimum model for each phenotype, a goodness-of-fit comparison was made based on Restricted Maximum Likelihood (REML) statistics using the Akaike Information Criterion (AIC).

Due to the expected annual effect on phenotypes and the missing data in our design (Additional file 11), a within-year model was performed for each year according to the following linear mixed model:

$$ {P}_{jk}=\mu +h+\phi +{O}_j+{G}_k+{e}_{jk} $$

Moreover, by considering a within-year model for each phenotype, we ensured the biological relevance of missing data replacement in the multi-year model allowed by the imputation due to the corh vcov structure.

Best unbiased linear predictors (BLUPs) were subsequently assessed from both within-year and multi-year models on *G* and *G* × *Y* random effects for the phenotypes *lgc* and *bs*. These predictors extracted from the multi-year model were then used as phenotypic input data for GWAS. A validation of the results was performed using the within-year BLUPs. In the case of *G* × *Y* BLUPs, imputed data were removed for missing individuals in each year in order to avoid statistical redundancy with the *G* BLUP prediction. REML estimates of genetic (${\sigma}_G^2$) and residual (${\sigma}_e^2$) variances were also computed from the multi-year model. Broad-sense heritability *H*^2^ for each phenotype per year was calculated as:

$$ {H}^2=\frac{\sigma_G^2}{\sigma_G^2+\frac{\sigma_e^2}{n}} $$

where *n* =3 is the number of replicates per accession.

### Genotyping and SNP marker data filtering

Genotyping and SNPs alignment were previously described in [30]. Briefly, the set of plants for GWAS was genotyped using NGS-Illumina HiSeq 2000/2500 sequencing with estimated depths between 15 to 25 folds depending on the accession. SNPs alignment was performed using the *Prunus persica* (*P. persica*) v1.0 reference genome and considering gene-space regions over the eight chromosome-level scaffold assemblies covering 99% of the genome [41]. Genotype assignment was performed according to an inference method based on maximum likelihood for estimating allele frequencies by using base counts at each position [42].

For our study, a minor allele frequency (MAF) threshold of 5% (according to [43]) was applied on the initial set of SNPs in order to remove rare variants and thus avoid false-positive associations. Then a filter on physical map position, discarding one of a pair of consecutive SNPs whose pairwise distance was less than 10 bp, was performed to limit the number of multiple statistical tests. After conducting all bioinformatic filters, a selection of 63,236 SNPs was kept for association study. Markers were named according to their physical positions on the genome, ‘LG1_72881’ for a SNP located on chromosome 1 at 72,881 bp, for example.

### Population stratification analysis

An additional filter based on LD pairwise pruning, that involved discarding markers for which squared correlation *r*^2^> 0.2 (window 50 - stepwise 5 SNPs), was executed in order to keep a set of 21,942 independent markers (under the assumption of linkage equilibrium) for inferring population structure.

The Admixture program [44] was used for computing maximum likelihood estimations of individual ancestry fractions while testing scenarii from k = 2 to k = 10 ancestral groups. The model choice criteria was based on minimization of the cross-validation error. In order to insure the reliability of the optimal choice of k, a complementary PCA analysis was performed on the genotype matrix. Inference of relatedness between individuals resulted in the calculation of an identity-by-state (IBS) allele sharing matrix from the same subset of 21,942 SNPs using the *emmax-kin* function implemented in the EMMAX (Efficient Mixed-Model Association eXpedited) program [45]. Both the ancestral proportion fraction matrix (Q) calculated according to the optimum model from Admixture and the IBS pairwise matrix between individuals (K) were used in GWAS to correct inflation of *p*-values due to stratification artefacts in the population.

### Linkage disequilibrium estimation

Pairwise LD from a sampling of 1000 markers per chromosome was computed using *r*^2^. A corrected procedure compacted in the *LDcorSV* R package [46] was used allowing removal of population stratification bias on LD. Considering a set of markers on each chromosome, both initial and corrected *r*^2^ estimates (${r}_{vs}^2$) were then plotted against physical distances in order to investigate intra-chromosomal LD decay according to the following model assuming drift-recombination equilibrium [47]${r}^2=\frac{1}{1+4 bd}+e$ where *r*^2^ is the square of loci correlation between a marker pair, *d* is the pairwise physical distance between the two markers, *b* is a decay coefficient calculated with least squares estimates in a non-linear regression (*nls* function in R software) and *e* refers to a residual estimate.

### Genome-wide association analysis

Cumulative effects of K and Q matrices over genotypic data were first tested with EMMAX [45] as a covariance genetic matrix and fixed ancestral covariates impacting expression of phenotypes. Quantile-quantile (Q-Q) plots were realized to select the best predictive model between K, Q and K + Q for each phenotype using the *qqman* R package [48]. Thus according to the best model choice, two supplementary mixed models were performed: a recently implemented multi-locus mixed-model [49] from the MLMM algorithm [50] (on both *G* and *G* × *Y* BLUPs from the multi-year model) and a multi-variate linear mixed-model (mvLMM) algorithm (between *lgc* and *bs G* BLUPs from the multi-year model) developed in GEMMA software [51]. Multi-locus models consist of forward stepwise mixed-model regressions with an increasing number of included markers (regressors) while re-estimating genetic and residual variance components at each step. The implementation from MLMM allows a more suitable handling of the so-called “high dimension issue” resulting from a low population size and a high number of possible regressors introduced into the model [49]. Instead of using a classical *p*-value threshold that considers only the number of multiple tests (number of SNPs), the model selection criterion is based on the calculation of a more permissive extended Bayesian Information Criterion eBIC [52]. *P*-values of marker-trait associations are given considering both the optimal model (with possibly several regressors) and each associated SNP as unique regressor avoiding p-value inflation due to forward regressor inclusion.

The multi-trait model takes advantage of the correlation structure between multiple phenotypes to increase power to detect not only pleiotropic genetic variants but also specific variants affecting only one of the correlated phenotypes [51]. Multi-testing correction on output *p*-values was performed considering False Discovery Rate (FDR) [53] control per chromosome (5% significance level).

For all significant associations, we computed the allelic effect *α* as (Minor allele mean – Major allele mean)/2 and the individual percentage of phenotypic variance explained (PVE). Furthermore, for each trait, strict sense heritability was calculated as the ratio ${h}^2=\frac{\sigma_{\sum SNP}^2}{\sigma_{\sum SNP}^2+{\sigma}_E^2}$ where ${\sigma}_{\sum SNP}^2$ is the additive genetic variance explained by all associated loci and ${\sigma}_E^2$ is the remaining variance linked to both dominance, epistasis genetic effects and non-genetic residual error. In order to ensure their reliability, all detected associations were subjected to further validations (ANOVA tests) on BLUPs from the within-year model.

Estimates of ${r}_{vs}^2$ around marker-trait-associated loci were computed to give a graphical representation of the pairwise LD using a modified version from the *snp.plotter* R package [54]. Moreover, performing genome-wide sampling of 1000 markers, ${r}_{vs}^2$ distributions were prospected considering both inter-chromosomal and candidate loci SNP pairs, and compared to intra-chromosomal scale in order to detect any specific linkages.

### Haplotype analysis

Groups of markers were constructed by considering all detected loci for each *G* and *G* × *Y* BLUP phenotype. Marker haplotypes were identified among all the accessions according to the genotypes on the candidate loci for each group of markers. Mean phenotypic distributions of the different haplotypes comprising at least two accessions were compared using HSD post-hoc tests (α = 5%) for the associated *G* BLUP phenotypes considering both multi-year and within-year models. For haplotypes represented by only one individual, mean value was compared with the significantly differing groups from the HSD tests. Favorable (and unfavorable) haplotypes were defined for each multi-year and within-year model *G* BLUP phenotype according to the following criteria: (i) showing a significantly lower (or upper) group mean value than all other haplotypes groups and (ii) with stable performances among years.

### Candidate gene identification

For each detected loci, candidate genes were identified and localized on the first and second version of the peach genome (*P. persica* v1.0 and v2.1, publicly available at https://www.rosaceae.org/species/prunus/all). Putative homolog protein annotations were obtained following Blastp searches upon the *Arabidopsis thaliana* (*A. thaliana*) genome using the TAIR10 database (https://www.arabidopsis.org/). SNP gene localization and effect on the protein sequence were determined using the JBrowse tool (https://www.rosaceae.org/tools/jbrowse) on the *P. persica* v1.0 genome and ORFfinder (https://www.ncbi.nlm.nih.gov/orffinder/), respectively. Then, the predicted impact of non-synonymous SNP on the biological function of the protein was evaluated with Provean v1.1.3 web-interface software (http://provean.jcvi.org/index.php) [55].

## Additional files

Additional file 1: Time series of degree-days over winter period in l’Amarine. 2013 to 2016 annual data from November 15th to March 31st. February month is delimited by the red arrows. (PDF 294 kb)

Additional file 2: Genetic (*G*) and genetic x year (*G* × *Y*) distributions of *lgc* and *bs* BLUPs. A. and B. *lgc* and *bs* density plots of *G* (red), (*G* × *Y*)_2013_ (green) and (*G* × *Y*)_2015_ (blue) adjusted BLUPs. C. Scatterplot showing the regression line between *lgc* and *bs G* BLUPs including 95% confidence interval. All BLUP values are represented on a ln(x + 1) scale. (TIFF 347 kb)

Additional file 3: Population Structure (Q) of the apricot core-collection based on data of 21,942 independent SNPs. Genotypic data pruned in order to keep markers with pairwise *r*^2^ values < 0.2. (A) Cross-validation estimation from k = 2 to k = 10 groups. Calculations performed using Admixture program [45]. (B) Barplot of Principal Component Analysis (PCA) eigenvalues from PC1 to PC73. The three first components explain 35.23% of the total variability. (C) Distribution of the ancestral fractions for each accession according to a structure divided into 3 groups: Central and Eastern Asia (yellow), Continental Europe (orange), Irano-Caucasian and Mediterranean Basin area (red). (PDF 169 kb)

Additional file 4: Relation between susceptibility phenotypes *lgc* and *bs G* BLUPs and ancestral fractions in the apricot core-collection. Ancestral fractions Q1 to Q3 are displayed as supplementary variables (in blue) in the PCA. The contribution of the first component reached 78.46% of the total variability. (PDF 97 kb)

Additional file 5: Linkage disequilibrium (LD) decay over physical position in the apricot core-collection. Pairwise *r*^2^ values were computed considering markers in a 1 kb-window. Estimated LD decay according to a non-linear regression is represented for each chromosome. Black curves show non-corrected estimates. Curves of corrected *r*^2^ estimates with relatedness K (red), structure Q (orange) and both K and Q (blue) are displayed. *r*^2^ mean and standard deviation values are indicated. *r*^2^=0.10 threshold are represented with dashed horizontal lines. (PDF 2325 kb)

Additional file 6: QQ (quantile-quantile) plots of the observed *p*-values distribution for *lgc* and *bs G* and *G* × *Y* BLUPs. Non-corrected generalized linear model (glm – green), glm accounting for structure Q (glm.Q – orange), linear mixed model correcting for relatedness K (emmax.K – blue) and both for K and Q (emmax.KQ – brown) output p-values are compared. The 95% confidence interval is indicated in grey. (PDF 387 kb)

Additional file 7:Effect of all detected loci using genome-wide multi-locus and multi-variate (mvLMM) mixed models on *lgc* and *bs G* and *G* × *Y* BLUPs. (A) Manhattan Plots displaying the –log10 (p-values) over physical positions in approximate 100 to 300 kb windows surrounding associations. Significance level determined with eBIC criterion. Detected SNPs were shown with red stars and overlapping candidate genes indicated with blue arrows. As an indication, Bonferroni threshold = −log10(p-value-thr) = 6.10. Pairwise LD heatmaps (${r}_{vs}^2\Big)$ were drawn within the genomic window around each candidate. (B) Boxplot displaying the allelic effect for the associated marker. For the loci detected with mvLMM on chromosome 7, the allelic effect is represented for both *lgc* and *bs G* BLUPs. (PDF 575 kb)

Additional file 8:Distribution of ${r}_{vs}^2$ unbiased estimates between intra-chromosomal, inter-chromosomal and detected loci random pairs of markers. Inter-chromosomal, intra-chromosomal and in between detected loci LD distributions are displayed in green, blue and red, respectively. Calculations performed considering 4.9E+ 06 intra-chromosomal and 4.3E+ 04 inter-chromosomal random pairs of markers. 99th percentile lines of ${r}_{vs}^2$ are drawn for intra-chromosomal and inter-chromosomal distributions. Correlations between candidate loci pairs LG6_15273858-LG5_5394803 (1st arrow) and LG5_5394803-LG5_4842835 (2nd arrow) are indicated. (TIFF 76 kb)

Additional file 9: Individual/multi-locus ANOVA and post-hoc mean comparison tests of all associated markers on *G* BLUPs from the within-year model. *** shows p-value below 0.001, ** between 0.001 and 0.01,* between 0.01 and 0.05, and ‘ns’ indicates non-significant p-values. Significant results are indicated in red. Results with p-values slightly over the 5% significance level (noted as (*.*)) are indicated in italic. SS = ratio sum of squares (factor/total variation). For ‘multi-locus’ ANOVA, strict-sense heritability estimates h^2^ are indicated. Comparison of means between groups were performed for individual locus-ANOVA only on phenotypes associated to a unique locus. AA/BB = homozygotes major/minor allele. AB = heterozygous. Letters indicate means differencing significantly between groups (HSD post-hoc test α = 5%). Group sizes are indicated between brackets for HSD post-hoc tests. (XLSX 15 kb)

Additional file 10: Marker haplotype composition of the apricot core-collection considering all detected loci. Groups of markers were constructed by considering all detected loci for each phenotype according to *G* and *G* × *Y* BLUPs. For markers, 0,1 and 2 are allelic codes according to minor allele dose. (i) Global *G* BLUPs from multi-year and within-year models associated with the haplotype. (ii) *P*-value of association between haplotype and trait. (iii) Number of accessions carrying the haplotype. (iv) Mean (± standard deviation) of associated trait per haplotype. (v) Post-hoc group mean comparison test (HSD α = 5%). HSD post-hoc tests were performed on *G* BLUPs from the multi-year and within-year models considering all haplotypes classes with *n* > 2. Green and red cases: extreme group values related to partially resistant and susceptible accessions for each phenotype. For haplotypes carried by only one individual, assessment was performed in comparison with significantly different groups from HSD post-hoc tests. Bold values show outliers in individual haplotypes from the extreme groups (values over the mean ± standard deviation of the extreme significant groups issued from HSD post-hoc tests). (XLSX 19 kb)

Additional file 11: Information of plant material used in the study: geographical origin, name and number of year repetitions through 2013 to 2016 phenotyping period. (XLSX 11 kb)

Additional file 12: Q ancestral covariates,*G*, *G* × *Y* BLUPs from the multi-year and *G* BLUPs from the within-year model of phenotypic data in the apricot core-collection. NA: missing values. Q1, Q2 and Q3 covariates are relative to ancestral fractions from (i) Central & Eastern Asia, (ii) Continental Europe and (iii) Irano-Caucasia & Mediterranean Basin, respectively. (XLSX 23 kb)

Additional file 13: Genotypic data of the 63,236 SNPs used for GWAS. MAF: Minor Allele Frequency. Genotypes are coded according to minor allele dose. 0/2 = homozygous major/minor allele. 1 = heterozygous. Markers names are indicated as LG[chromosome number]_[physical position in bp]. (XLSX 17724 kb)

## Abbreviations

*h*^2^: Strict-sense heritability
*H*^2^: Broad-sense heritability
ABA: Abscisic acid
AIC: Akaike information criterion
ANOVAq: Analysis of variance
BLUP: Best linear unbiased predictor
bp: Base pair
*bs*: Superficial browning
CDS: Coding sequences
corh: Correlation
eBIC: Extended bayesian information criterion
FDR: False discovery rate
G: Genetic
glm: Generalized linear model
GWAS: Genome-wide association study
JA: Jasmonic acid
K: Kinship
LD: Linkage disequilibrium
LG: Linkage group
*lgc*: Canker length
MAF: Minor-allele frequency
MAS: Marker-assisted selection
MLE: Maximum likelihood estimation
mvLMM: Multi variate linear mixed model
NA: Non-applicable
PCA: Principal component analysis
*Ps*: *Pseudomonas syringae*
PVE: Percentage of variation explained
Q: Population structure
QQ-plots: Quantile-quantile plots
QTL: Quantitative trait loci
REML: Restricted maximum likelihood
SA: Salicylic acid
SNP: Single-nucleotide polymorphism
vcorr: Variance-covariance
Y: Year
