# Supplementary figures and images for: Genome-wide association multi-locus and multi-variate linear mixed models reveal two linked loci with major effects on partial resistance of apricot to bacterial canker
Source: BMC Plant Biol. 2019 Jan 21;19:31. doi: 10.1186/s12870-019-1631-3 (PMC6341767; doi:10.1186/s12870-019-1631-3)

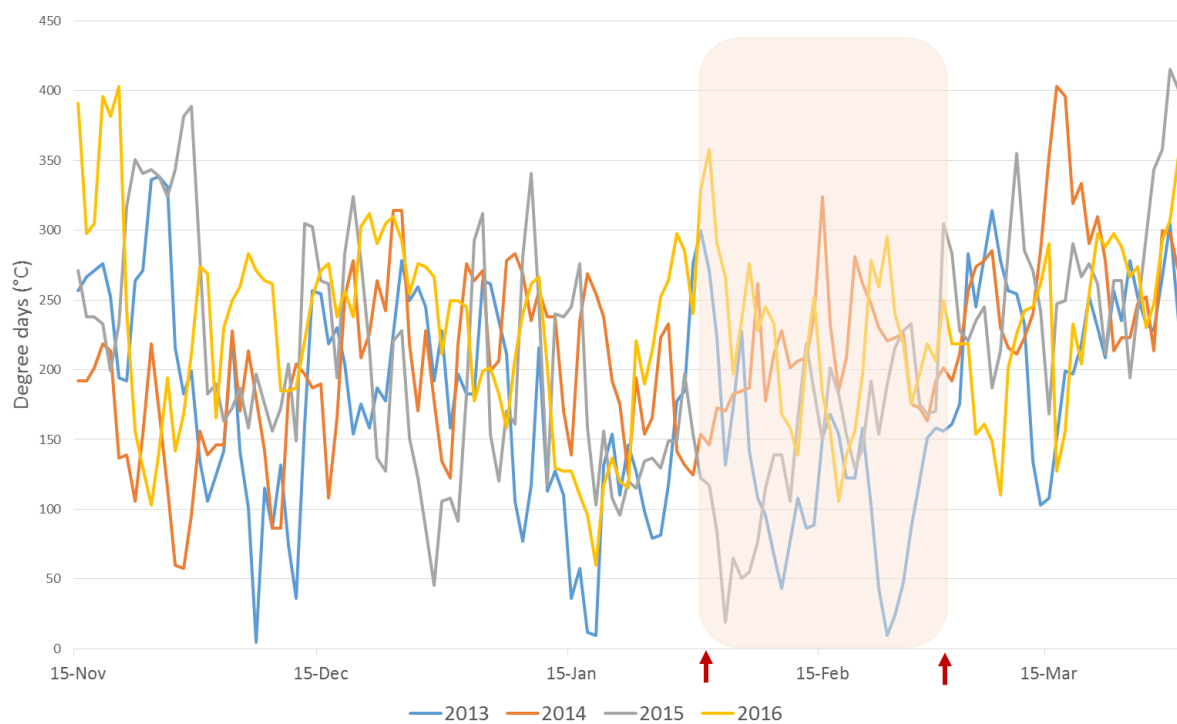

Supplement: Supplementary file 1 — Time series of degree-days over winter period in l’Amarine. 2013 to 2016 annual data from November 15th to March 31st. February month is delimited by the red arrows. (PDF 294 kb) [file 12870_2019_1631_MOESM1_ESM.pdf]

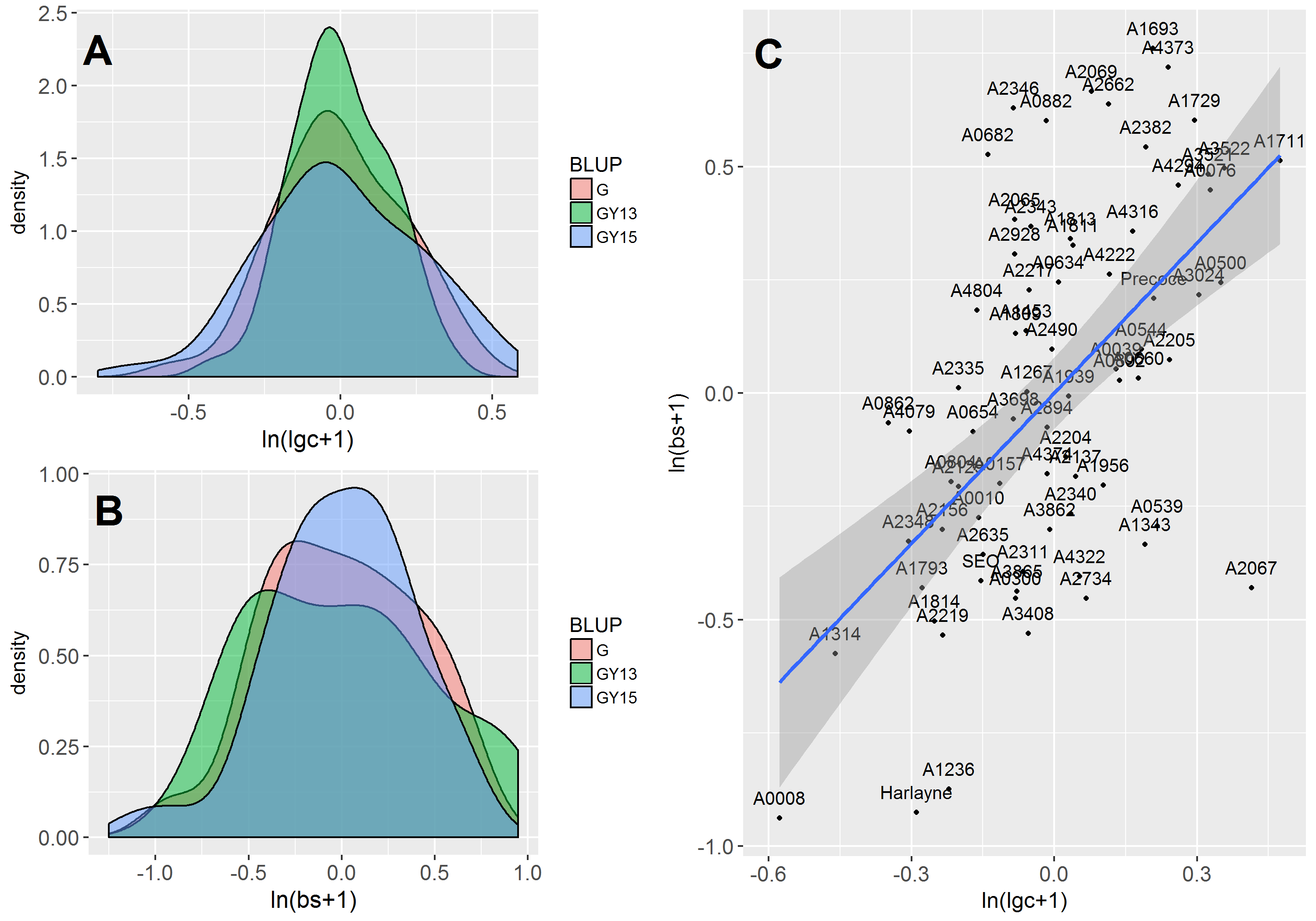

Supplement: Supplementary file 2 — Genetic (G) and genetic x year (G × Y) distributions of lgc and bs BLUPs. A. and B. lgc and bs density plots of G (red), (G × Y)2013 (green) and (G × Y)2015 (blue) adjusted BLUPs. C. Scatterplot showing the regression line between lgc and bs G BLUPs including 95% confidence interval. All BLUP values are represented on a ln(x + 1) scale. (TIFF 347 kb) [file 12870_2019_1631_MOESM2_ESM.tiff]

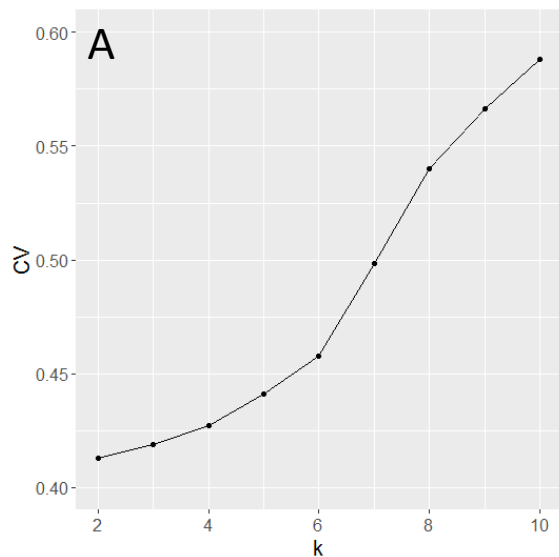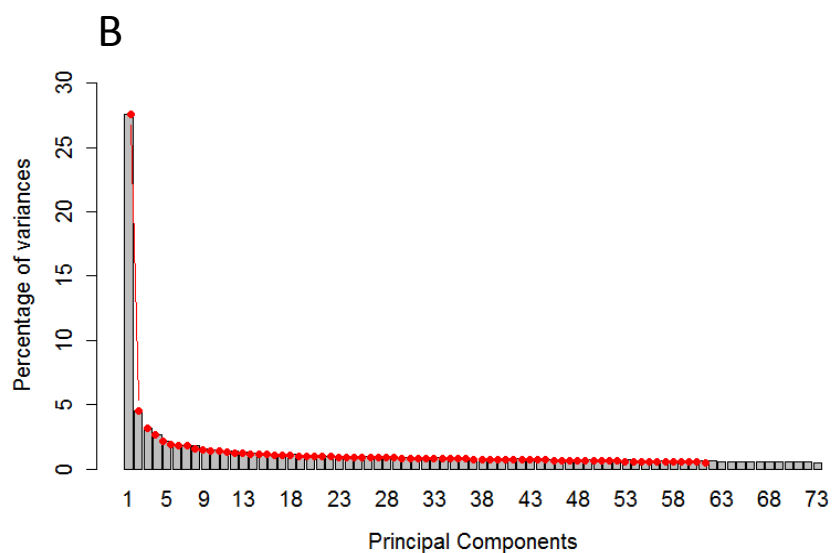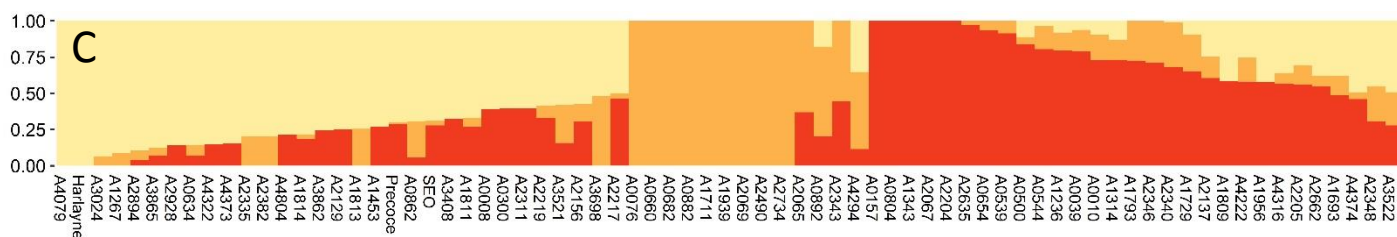

Supplement: Supplementary file 3 — Population Structure (Q) of the apricot core-collection based on data of 21,942 independent SNPs. Genotypic data pruned in order to keep markers with pairwise r2 values < 0.2. (A) Cross-validation estimation from k = 2 to k = 10 groups. Calculations performed using Admixture program [45]. (B) Barplot of Principal Component Analysis (PCA) eigenvalues from PC1 to PC73. The three first components explain 35.23% of the total variability. (C) Distribution of the ancestral fractions for each accession according to a structure divided into 3 groups: Central and Eastern Asia (yellow), Continental Europe (orange), Irano-Caucasian and Mediterranean Basin area (red). (PDF 169 kb) [file 12870_2019_1631_MOESM3_ESM.pdf]

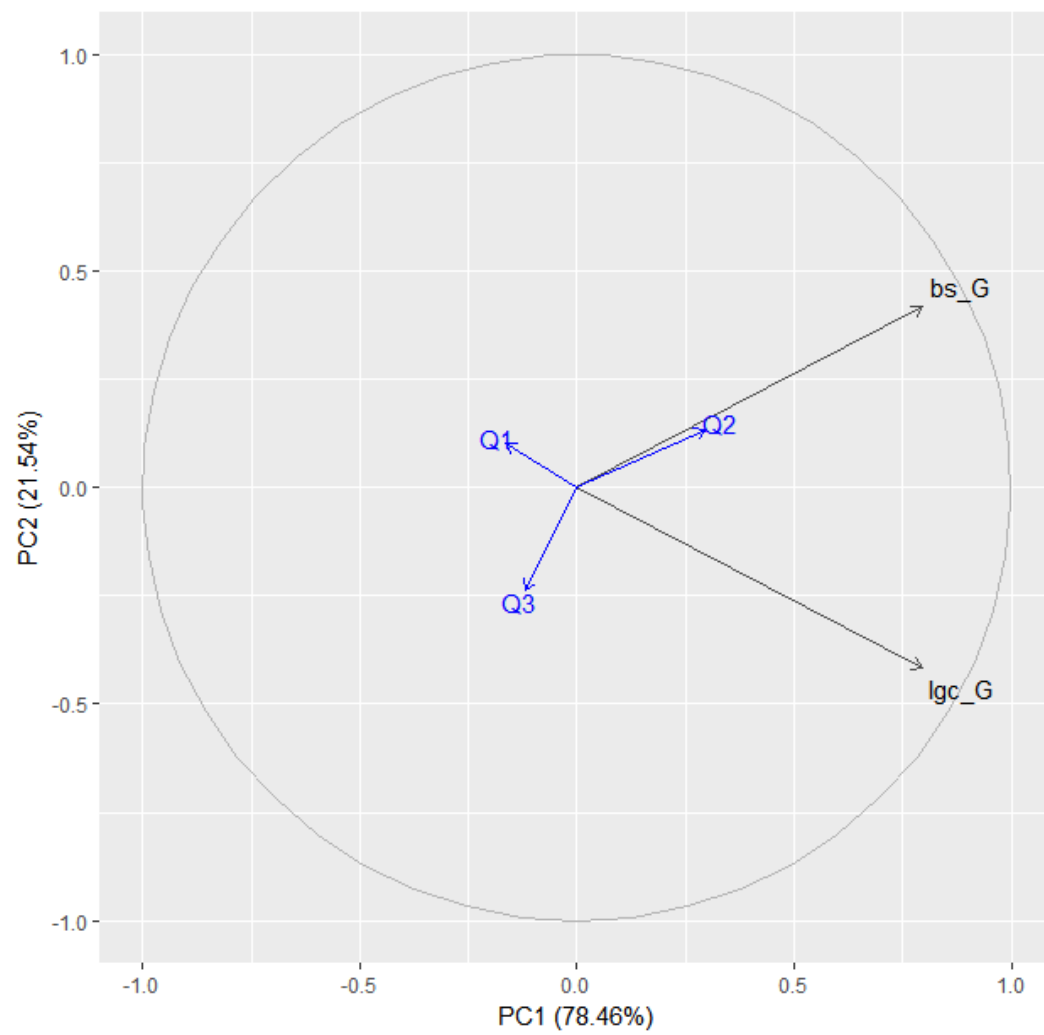

Supplement: Supplementary file 4 — Relation between susceptibility phenotypes lgc and bs G BLUPs and ancestral fractions in the apricot core-collection. Ancestral fractions Q1 to Q3 are displayed as supplementary variables (in blue) in the PCA. The contribution of the first component reached 78.46% of the total variability. (PDF 97 kb) [file 12870_2019_1631_MOESM4_ESM.pdf]

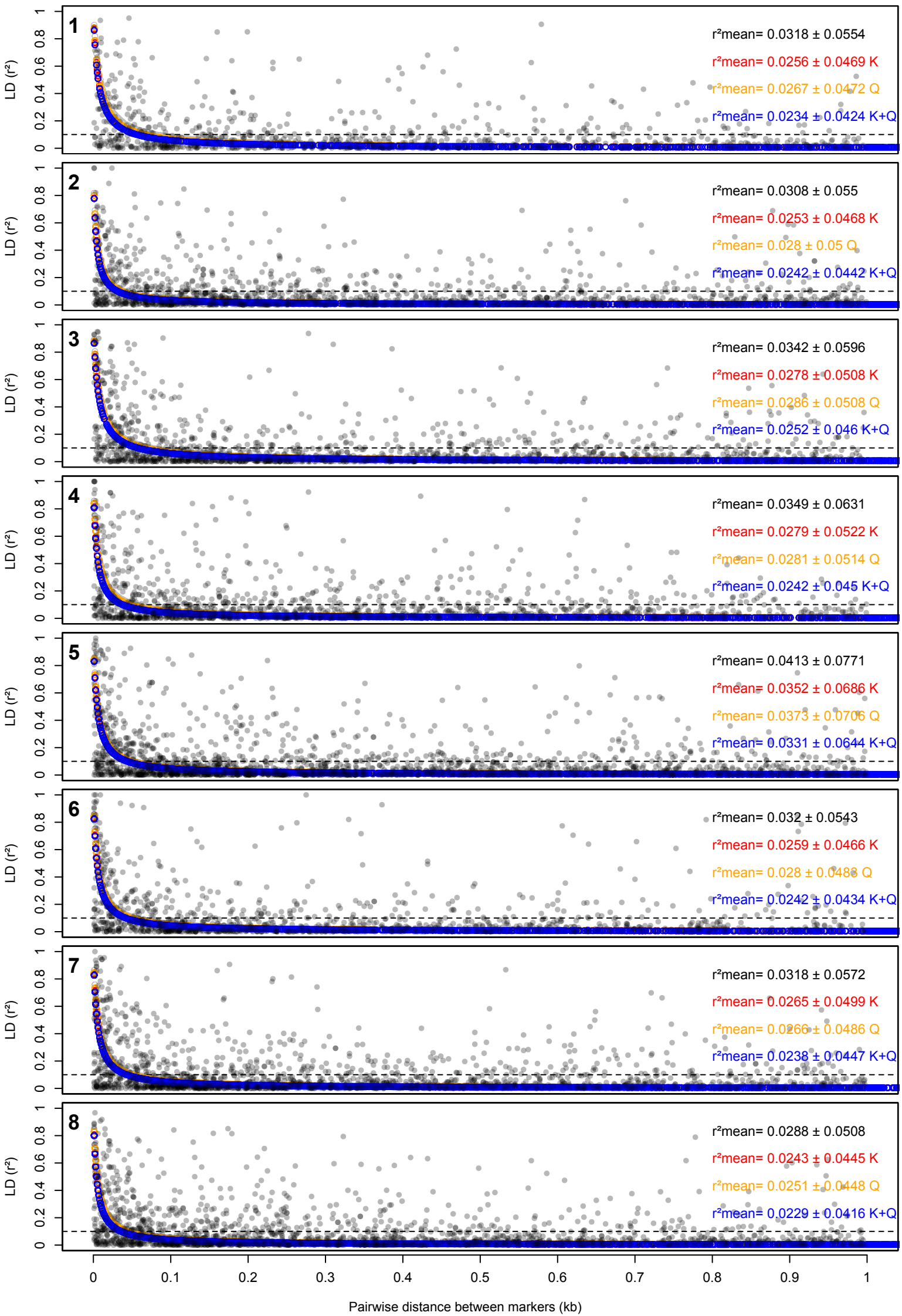

Supplement: Supplementary file 5 — Linkage disequilibrium (LD) decay over physical position in the apricot core-collection. Pairwise r2 values were computed considering markers in a 1 kb-window. Estimated LD decay according to a non-linear regression is represented for each chromosome. Black curves show non-corrected estimates. Curves of corrected r2 estimates with relatedness K (red), structure Q (orange) and both K and Q (blue) are displayed. r2 mean and standard deviation values are indicated. r2=0.10 threshold are represented with dashed horizontal lines. (PDF 2325 kb) [file 12870_2019_1631_MOESM5_ESM.pdf]

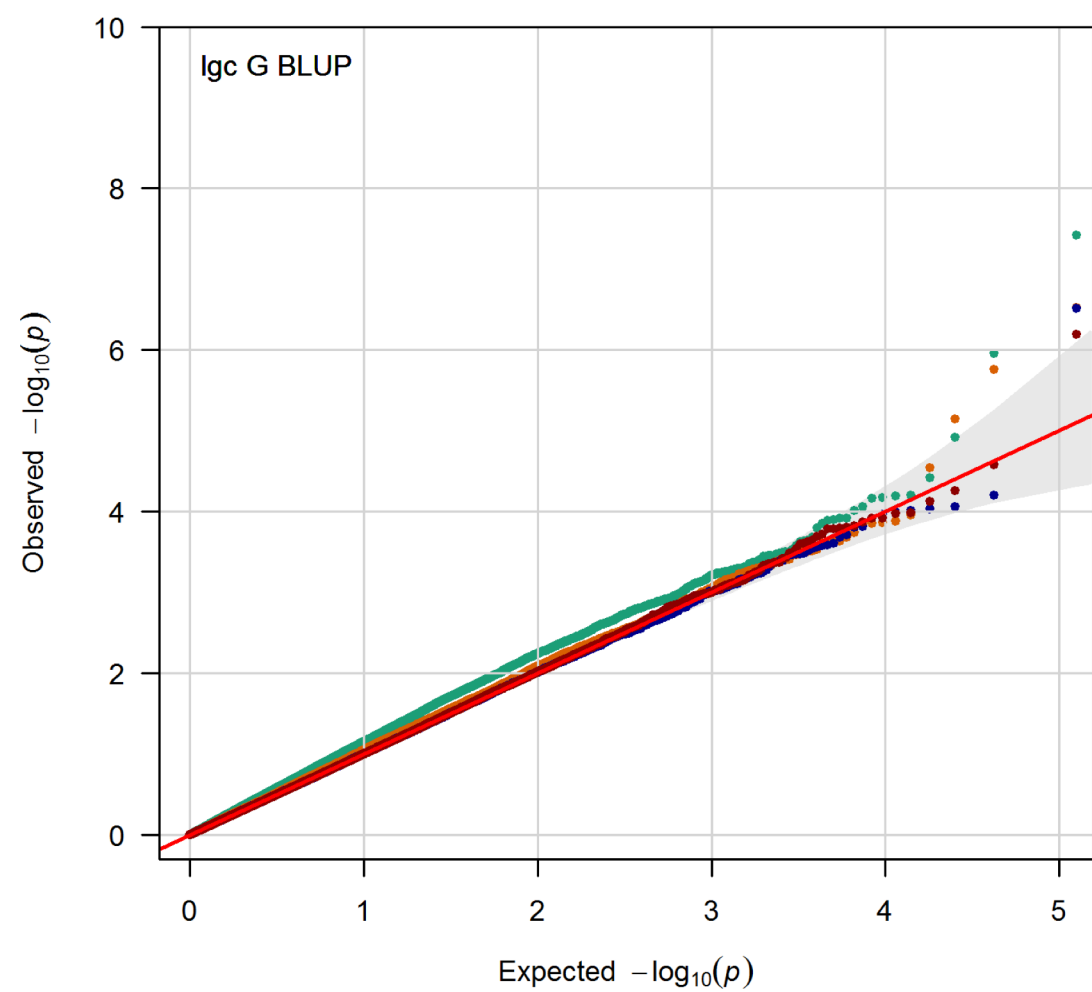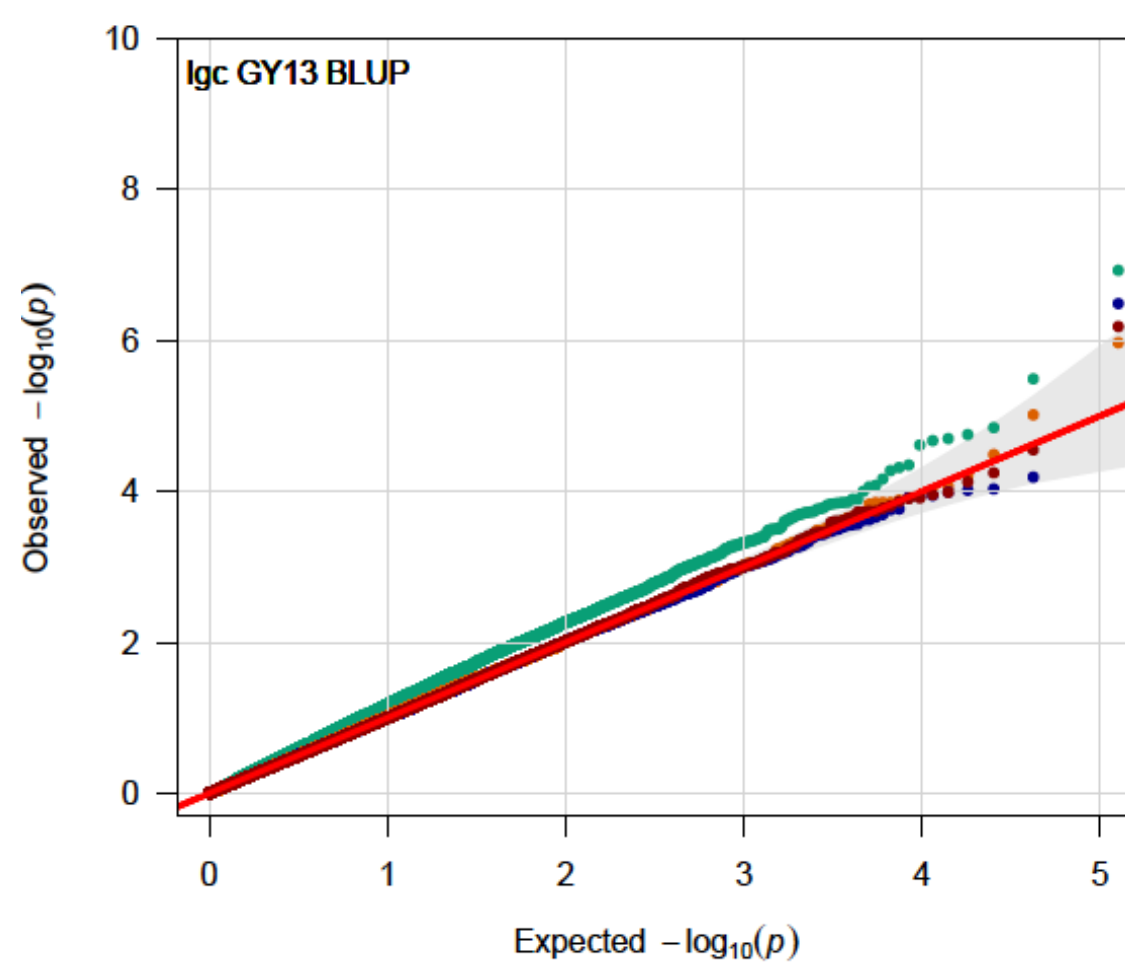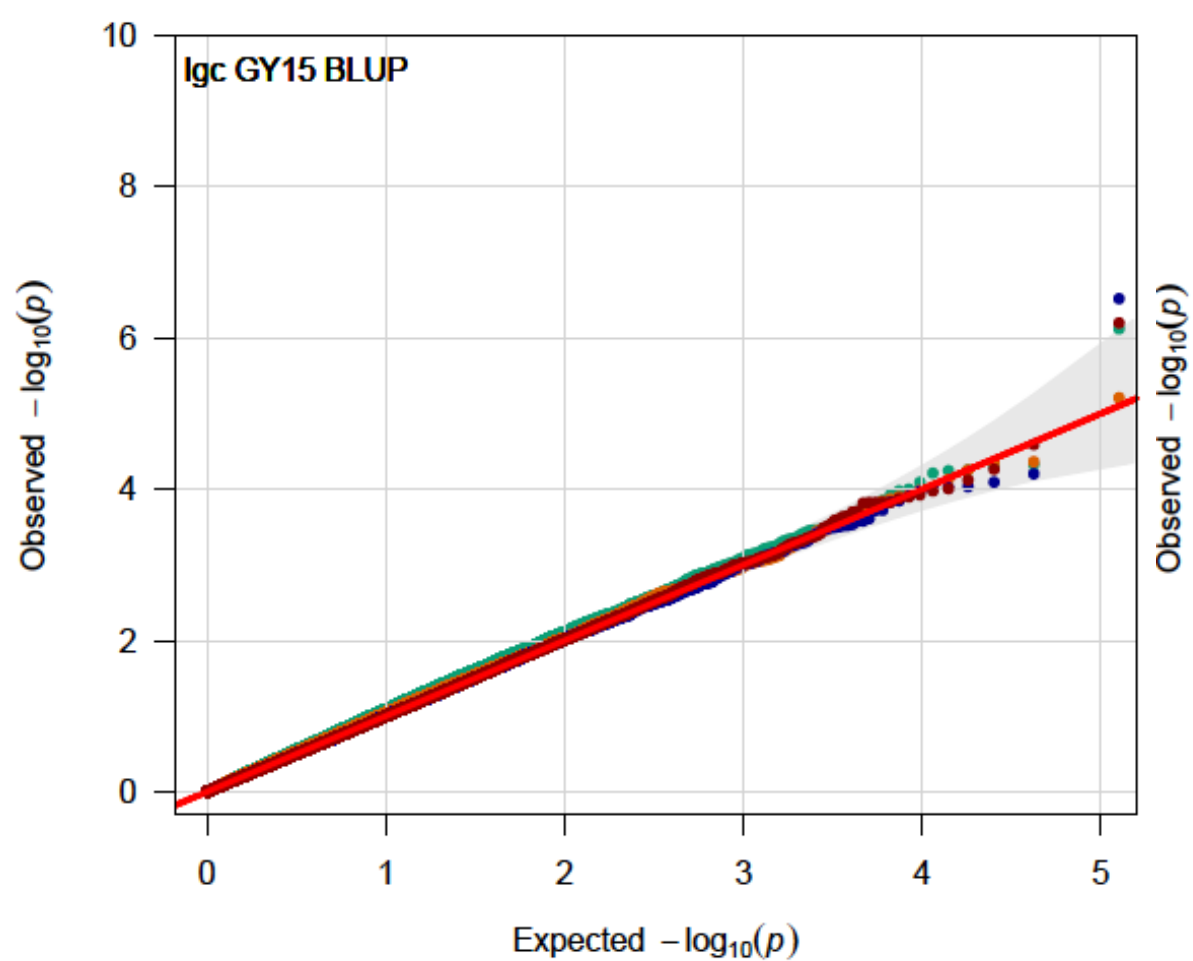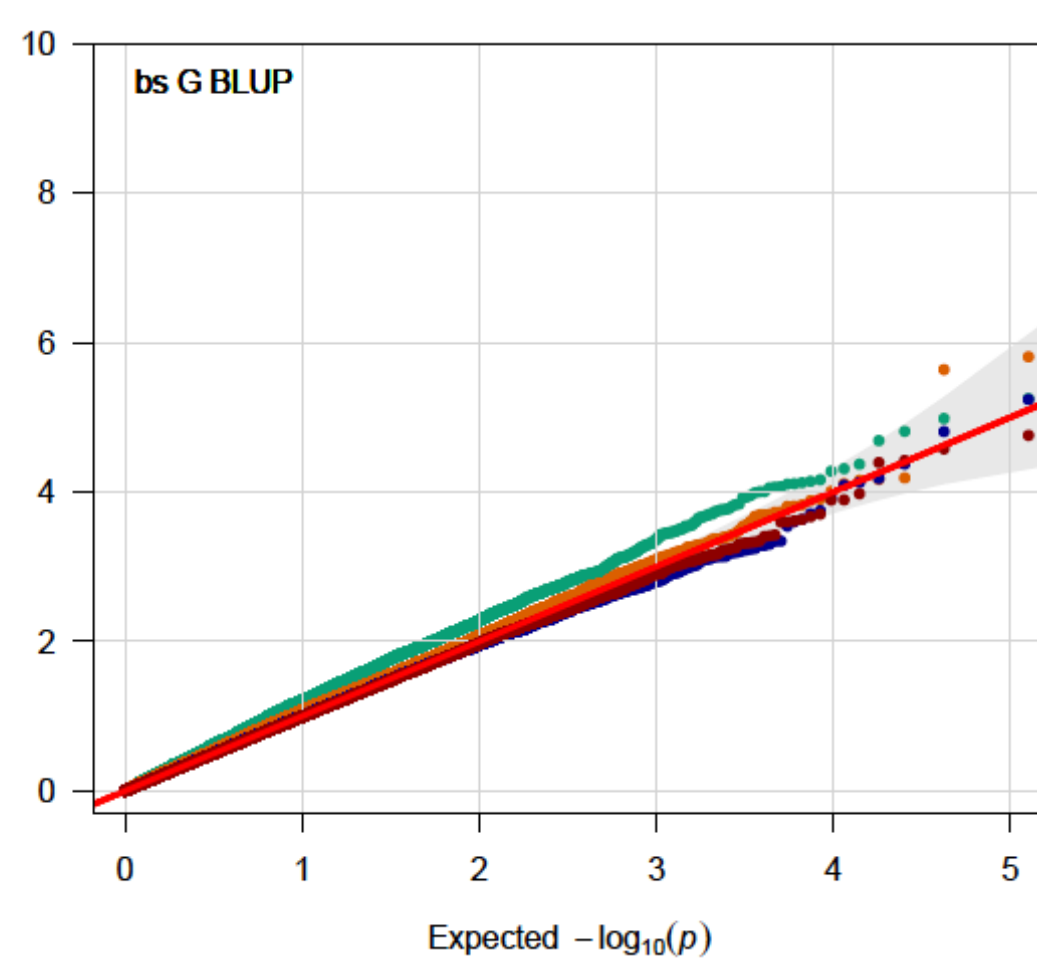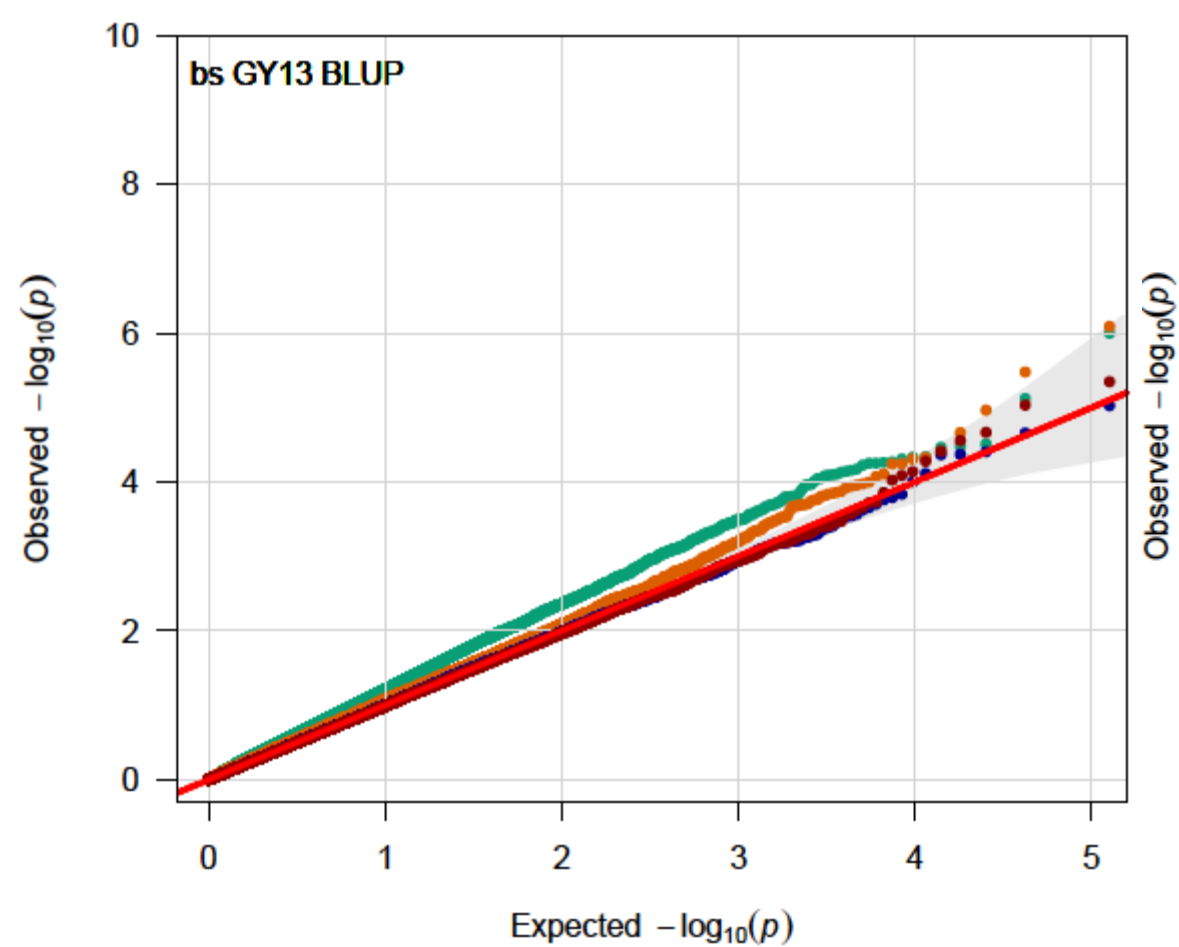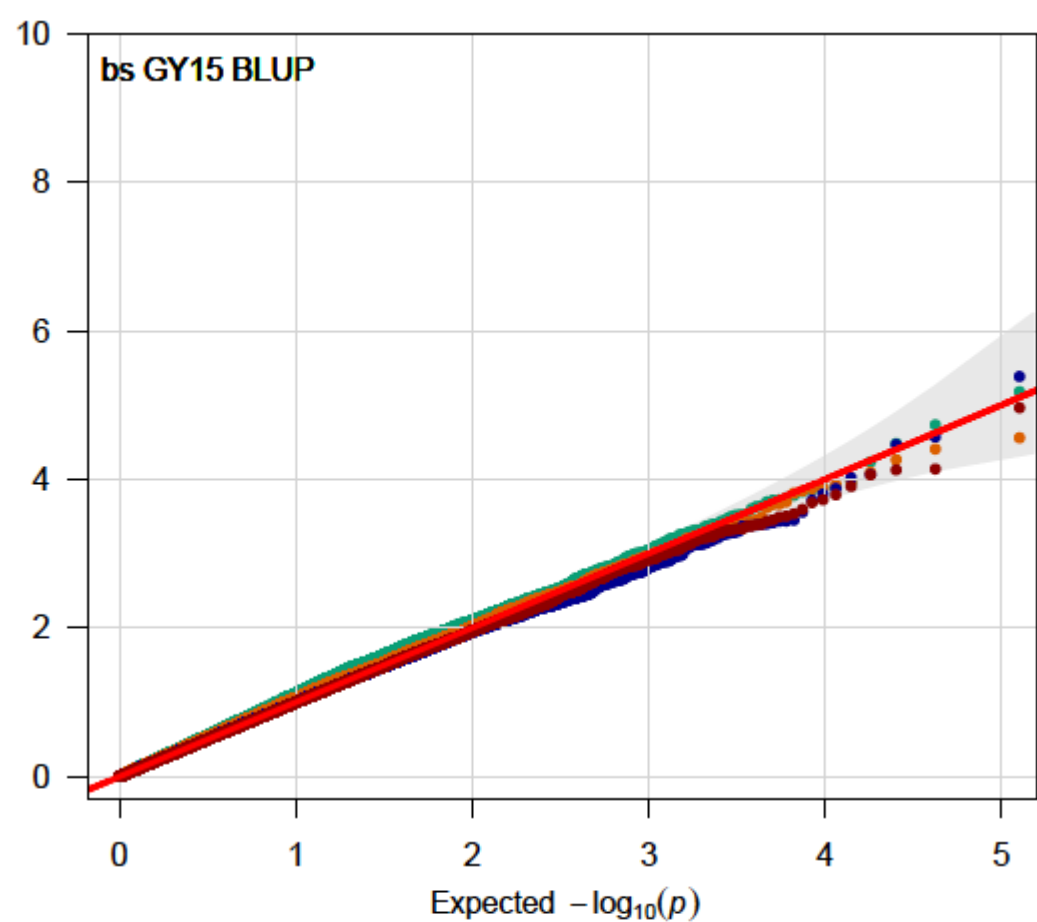

—●— glm    —●— glm.Q    —●— emmax.K    —●— emmax.KQ

Supplement: Supplementary file 6 — QQ (quantile-quantile) plots of the observed p-values distribution for lgc and bs G and G × Y BLUPs. Non-corrected generalized linear model (glm – green), glm accounting for structure Q (glm.Q – orange), linear mixed model correcting for relatedness K (emmax.K – blue) and both for K and Q (emmax.KQ – brown) output p-values are compared. The 95% confidence interval is indicated in grey. (PDF 387 kb) [file 12870_2019_1631_MOESM6_ESM.pdf]

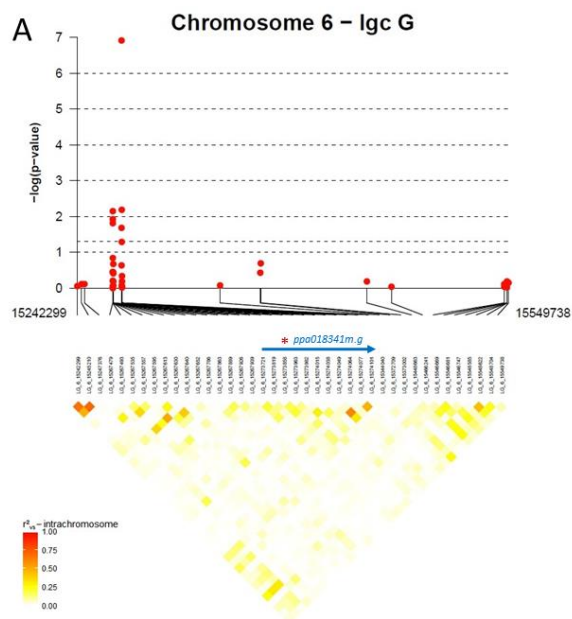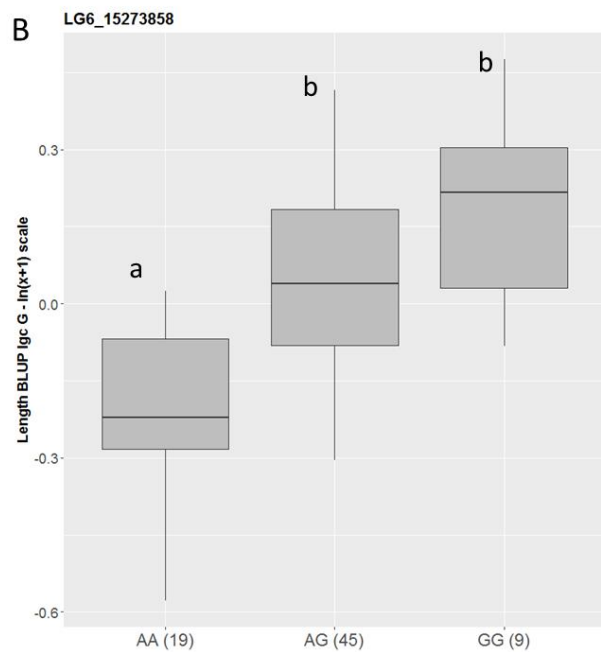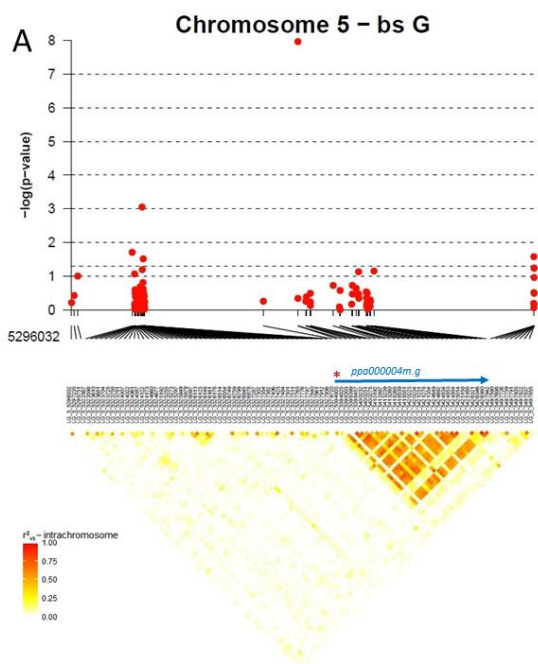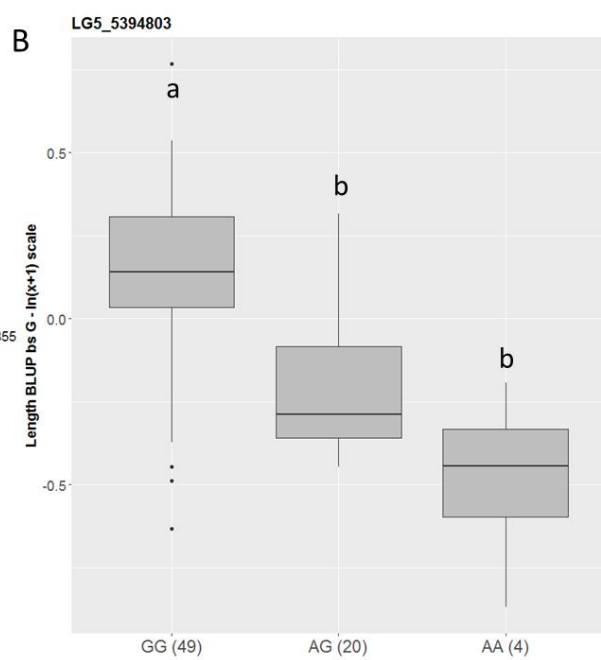

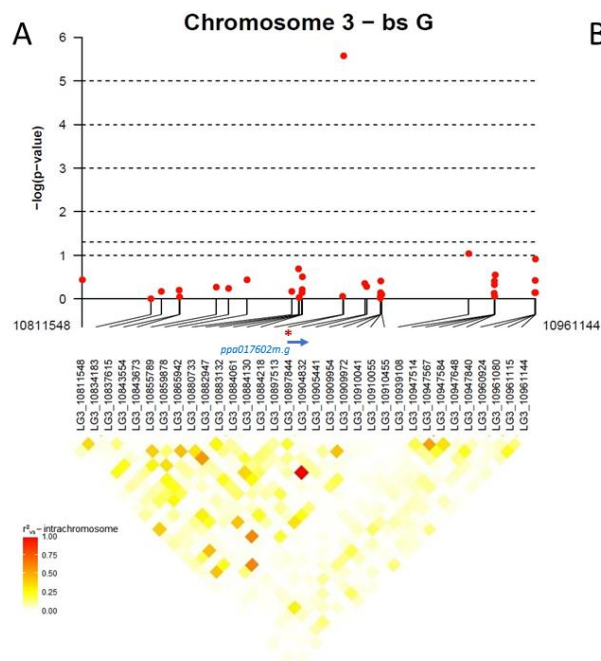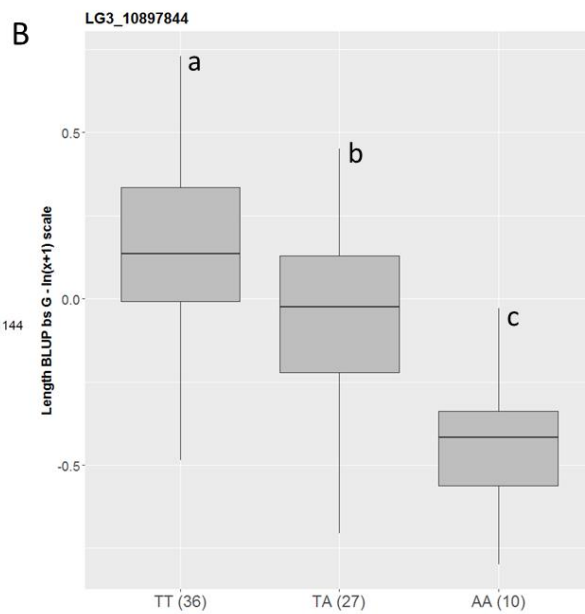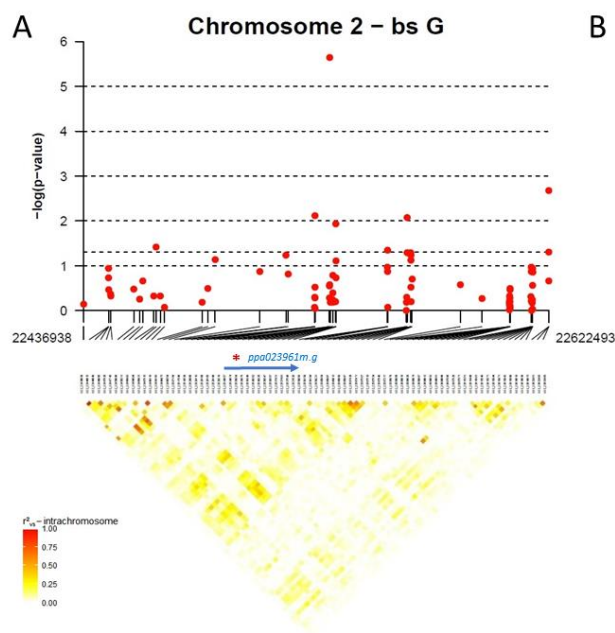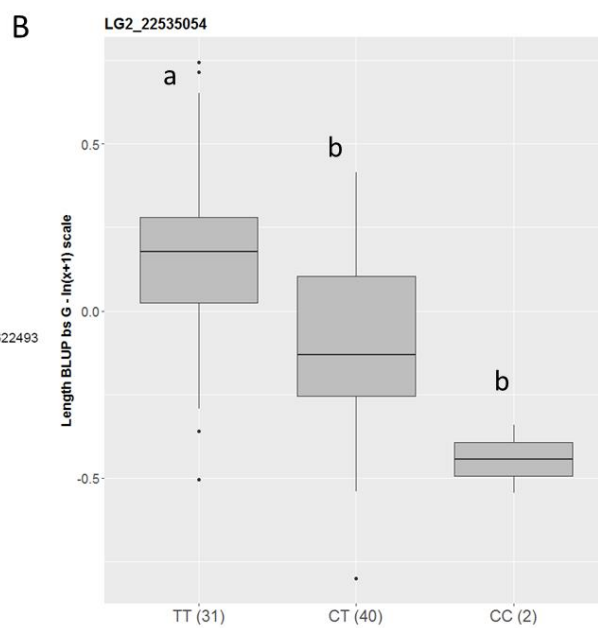

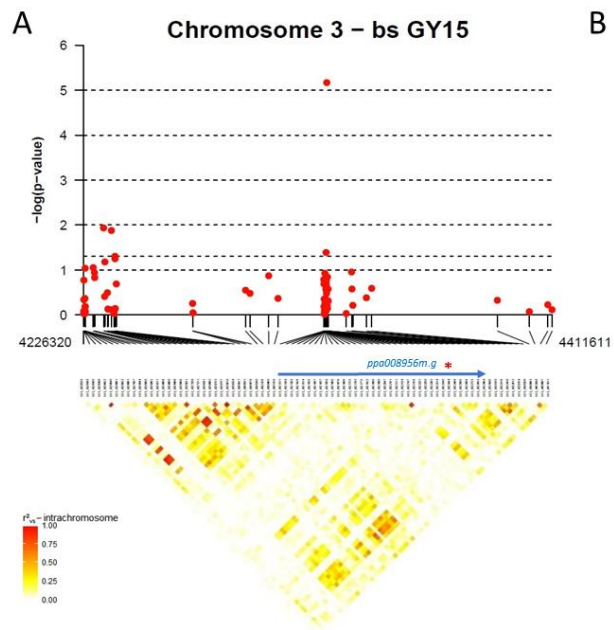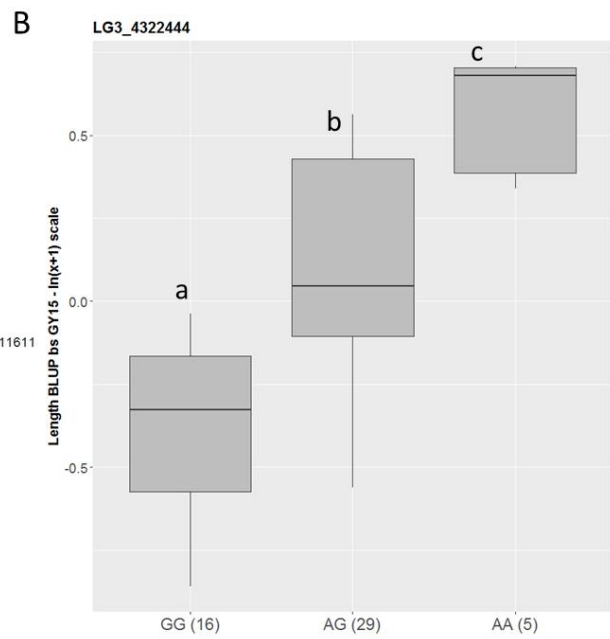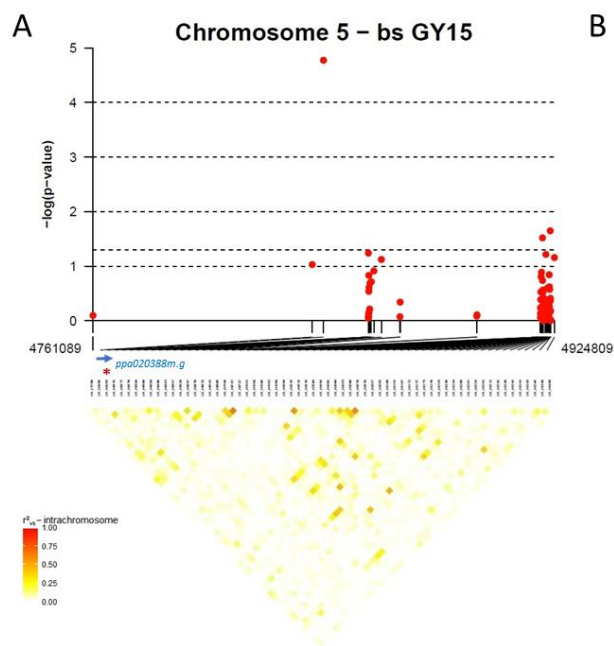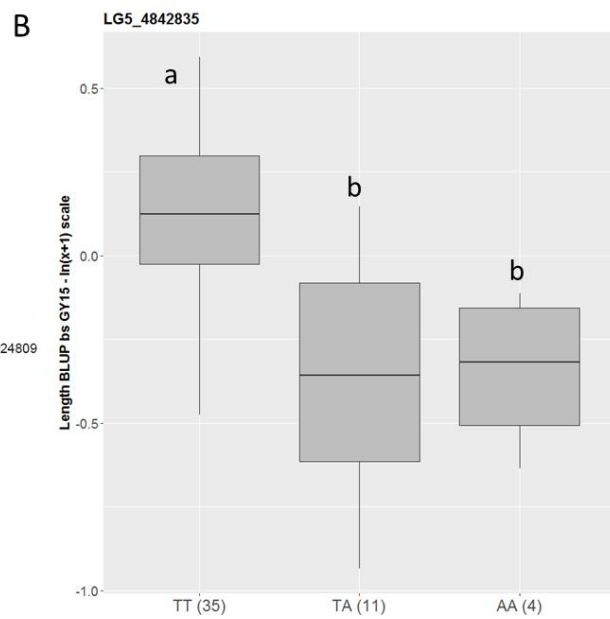

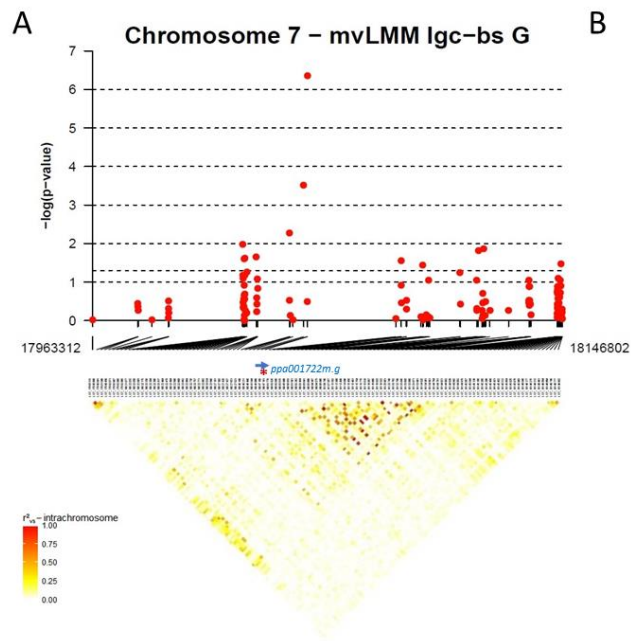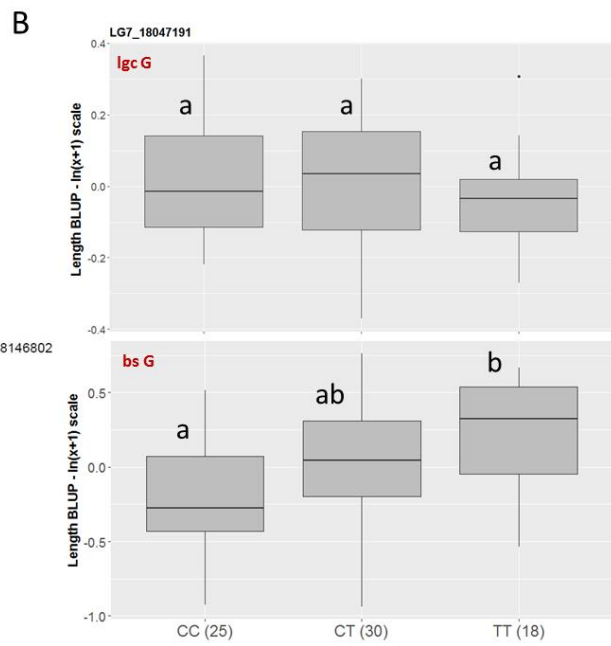

Supplement: Supplementary file 7 — Effect of all detected loci using genome-wide multi-locus and multi-variate (mvLMM) mixed models on lgc and bs G and G × Y BLUPs. (A) Manhattan Plots displaying the –log10 (p-values) over physical positions in approximate 100 to 300 kb windows surrounding associations. Significance level determined with eBIC criterion. Detected SNPs were shown with red stars and overlapping candidate genes indicated with blue arrows. As an indication, Bonferroni threshold = −log10(p-value-thr) = 6.10. Pairwise LD heatmaps (${r}_{vs}^2\Big)$rvs2) were drawn within the genomic window around each candidate. (B) Boxplot displaying the allelic effect for the associated marker. For the loci detected with mvLMM on chromosome 7, the allelic effect is represented for both lgc and bs G BLUPs. (PDF 575 kb) [file 12870_2019_1631_MOESM7_ESM.pdf]

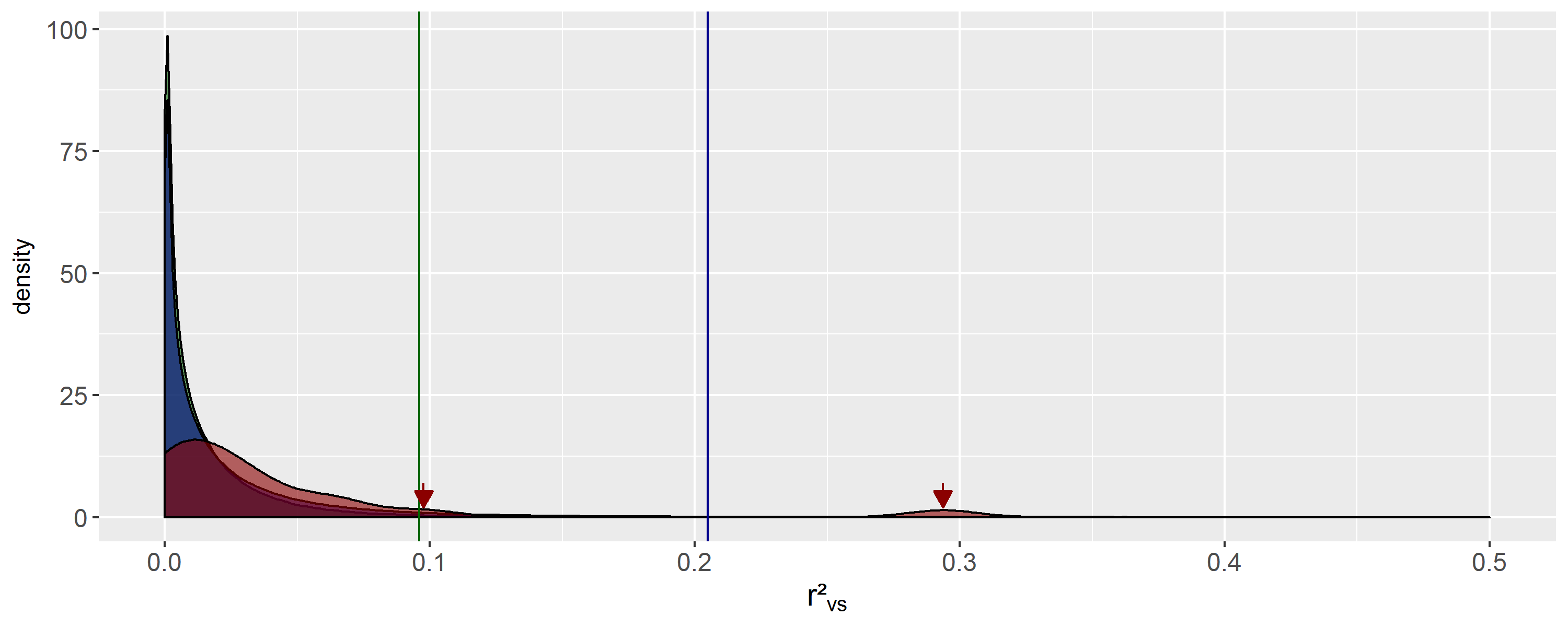

Supplement: Supplementary file 8 — Distribution of ${r}_{vs}^2$rvs2 unbiased estimates between intra-chromosomal, inter-chromosomal and detected loci random pairs of markers. Inter-chromosomal, intra-chromosomal and in between detected loci LD distributions are displayed in green, blue and red, respectively. Calculations performed considering 4.9E+ 06 intra-chromosomal and 4.3E+ 04 inter-chromosomal random pairs of markers. 99th percentile lines of ${r}_{vs}^2$rvs2 are drawn for intra-chromosomal and inter-chromosomal distributions. Correlations between candidate loci pairs LG6_15273858-LG5_5394803 (1st arrow) and LG5_5394803-LG5_4842835 (2nd arrow) are indicated. (TIFF 76 kb) [file 12870_2019_1631_MOESM8_ESM.tiff]
